# Network Physiology: How Organ Systems Dynamically Interact

**DOI:** 10.1371/journal.pone.0142143

**Published:** 2015-11-10

**Authors:** Ronny P. Bartsch, Kang K. L. Liu, Amir Bashan, Plamen Ch. Ivanov

**Affiliations:** 1 Department of Physics, Bar-Ilan University, Ramat Gan, 52900, Israel; 2 Department of Physics, Boston University, Boston, MA 02215, United States of America; 3 Department of Neurology, Beth Israel Deaconess Medical Center and Havard Medical School, Boston, MA 02115, United States of America; 4 Harvard Medical School and Channing Division of Network Medicine, Brigham and Women’s Hospital, Boston, MA 02115, United States of America; 5 Harvard Medical School and Division of Sleep Medicine, Brigham and Women’s Hospital, Boston, MA 02115, United States of America; 6 Institute of Solid State Physics, Bulgarian Academy of Sciences, Sofia 1784, Bulgaria; University of Maribor, SLOVENIA

## Abstract

We systematically study how diverse physiologic systems in the human organism dynamically interact and collectively behave to produce distinct physiologic states and functions. This is a fundamental question in the new interdisciplinary field of Network Physiology, and has not been previously explored. Introducing the novel concept of Time Delay Stability (TDS), we develop a computational approach to identify and quantify networks of physiologic interactions from long-term continuous, multi-channel physiological recordings. We also develop a physiologically-motivated visualization framework to map networks of dynamical organ interactions to graphical objects encoded with information about the coupling strength of network links quantified using the TDS measure. Applying a system-wide integrative approach, we identify distinct patterns in the network structure of organ interactions, as well as the frequency bands through which these interactions are mediated. We establish first maps representing physiologic organ network interactions and discover basic rules underlying the complex hierarchical reorganization in physiologic networks with transitions across physiologic states. Our findings demonstrate a direct association between network topology and physiologic function, and provide new insights into understanding how health and distinct physiologic states emerge from networked interactions among nonlinear multi-component complex systems. The presented here investigations are initial steps in building a first atlas of dynamic interactions among organ systems.

## Introduction

The human organism comprises diverse organ systems. Each organ system has its own complex structural organization and regulatory mechanisms that lead to complex transient, intermittent and nonlinear behavior. Medical specialists traditionally focus on a single physiological system: cardiologists examine the heart and consider ECG signals; pulmonologists check lung structure and function and probe respiratory patterns; brain neurologists utilize MRI and brain wave EEG signals. However, the human organism is an integrated network, where multi-component organ systems continuously interact through various feedback mechanisms and across different spatio-temporal scales to optimize and coordinate their function. Coordinated interactions of organ systems are essential to maintain health and to generate distinct physiologic states, e.g., wake and sleep; light and deep sleep; consciousness and unconsciousness. Altering or disrupting organ communications can lead to dysfunction of individual systems or to collapse of the entire organism, e.g., fever, hypertension, pneumonia, coma, multiple organ failure. Yet, despite the importance to understanding basic physiologic functions and the broad clinical relevance, we know almost nothing about the nature of the dynamical interactions between diverse organ systems and their collective role as an integrated network in maintaining health.

Understanding integrated physiologic function as emergent phenomena from complex interactions among diverse organ systems is the main focus of a new field, Network Physiology [1–3]. Network Physiology aims to develop theoretical framework and a system-wide network approach to understand how horizontal integration of physiological systems, each with its own complex structure and mechanisms of regulation, leads to global behavior and distinct physiologic functions at the organism level. The need of new analytic tools and a theoretical framework to address the special class of dynamical networks encountered in physiological systems has recently generated a broad interest in the community of physicists, applied mathematicians, neuroscientists and physiologists.

In recent years, investigations in the field of Network Physiology were extended from organ interactions to encompass horizontal integration of physiological systems at different levels in the organism—ranging from protein dynamics in wound healing processes at the sub-cellular level [4], to networks of neuronal group interactions at the cellular level [5–8], network communications in distinct tissues [9], and up to the level of network interactions within and among organ systems [1, 2, 10–14]. Further, empirical studies were accompanied by theoretical modeling efforts with the aim to understand emergent network dynamics out of coupled and synchronized nonlinear oscillators [15–18] to link microscopic and macroscopic scales in complex network dynamics as encountered in Network Physiology.

Here, we focus on understanding the nature of interactions between a number of key physiologic organ systems, including the cerebral, cardiac, respiratory, ocular and motor system. These are diverse and complex systems, with their own regulatory mechanisms, characteristic time scales, and with very different types of output signals. We investigate whether each physiologic state is associated with a specific network of interactions among organ systems that is characterized by a given topology, node connectivity, number and strength of network links. We propose a novel network approach based on the concept of Time Delay Stability (See Section Methods), which can identify direct and indirect pathways of interaction among physiologic systems—information which can not be deduced from the analysis of pair-wise coupling alone.

It is instrumental to determine how the output dynamics of one system can be affected by changes in the dynamics of other systems via the network of interactions. To this end, we study how the synchronization and coordination among physiologic systems collectively change in time. In particular, we track the evolution of network topology and structure with transitions from one physiologic state to another (e.g., sleep stages). Previous studies have demonstrated that basic linear and non-linear characteristics of the output dynamics of key organ systems under neural regulation change with transitions across different physiologic states such as rest and exercise [19], wake and sleep [20], different sleep stages [21–25], and circadian phases [26–28]. Thus, we investigate whether transitions in physiologic state would lead to changes in the network dynamics of physiological organ systems and to specific hierarchical reorganization of physiologic network characteristics. Further, we hypothesize that such structured reorganizations may simultaneously occur globally in the entire network as well as at the level of individual organ systems (network nodes). Finding robust patterns in the reorganization of these physiologic networks would reveal new dynamical aspects of basic physiologic regulation and would help uncover previously unknown associations between network structure of organ interactions and distinct physiologic functions.

To map physiologic coupling among organ systems onto graphical objects, and to translate complex dynamical interactions to physiologic networks, we develop a physiologically motivated visualization framework (Section Methods). Such network maps may have broad clinical implications and can serve as blueprints of different physiologic states under healthy and pathologic conditions. The presented results are initial steps that would ultimately lead to the development of a first Atlas of dynamical interactions between physiological systems in the human organism.

## Data

We analyze continuously recorded multi-channel physiological data obtained from 36 healthy young subjects (18 female, 18 male, with ages between 20–40, average 29 years) during night-time sleep (average record duration is 7.8 h). We focus on physiological dynamics during sleep as sleep stages are well-defined physiological states, and external influences due to physical activity or sensory inputs are reduced during sleep. Sleep stages are scored in 30 s epochs by sleep lab technicians based on standard criteria [29]. Specifically, we analyze EEG data from six scalp locations (frontal left–Fp1, frontal right–Fp2, central left–C3, central right–C4, occipital left–O1, and occipital right–O2), the electrocardiogram (ECG), respiration, the electrooculogram (EOG), and the electromyogram (EMG) of the chin and leg. In order to compare these very different signals with each other and to study interrelations between them, we extract the following time series from the raw signals: the spectral power of seven frequency bands of the EEG in moving windows of 2 s with a 1 s overlap: *δ* (0–4 Hz), *θ* (4–8 Hz), *α* (8–12 Hz), *σ* (12–16 Hz), *β* (16–20 Hz), *γ*_1_ (20–34Hz) and *γ*_2_ (34–100 Hz); the variance of the EOG and EMG signals in moving windows of 2 s with a 1 s overlap; heartbeat RR intervals and interbreath intervals are both re-sampled to 1 Hz (1 s bins) after which values are inverted to obtain heart rate and respiratory rate. Thus, all time series have the same time resolution of 1 s before the analysis.

The data we used in this work are pre-existing multi-channel physiologic recordings from EU SIESTA databases [30]. All participants provided written informed consent. The research protocol was approved (protocol number 3380X) by the Institutional Review Boards of Boston University (Boston, MA, USA) and was conducted according to the principles expressed in the Declaration of Helsinki.

## Methods

### Time delay stability (TDS)

**Fig 1:**
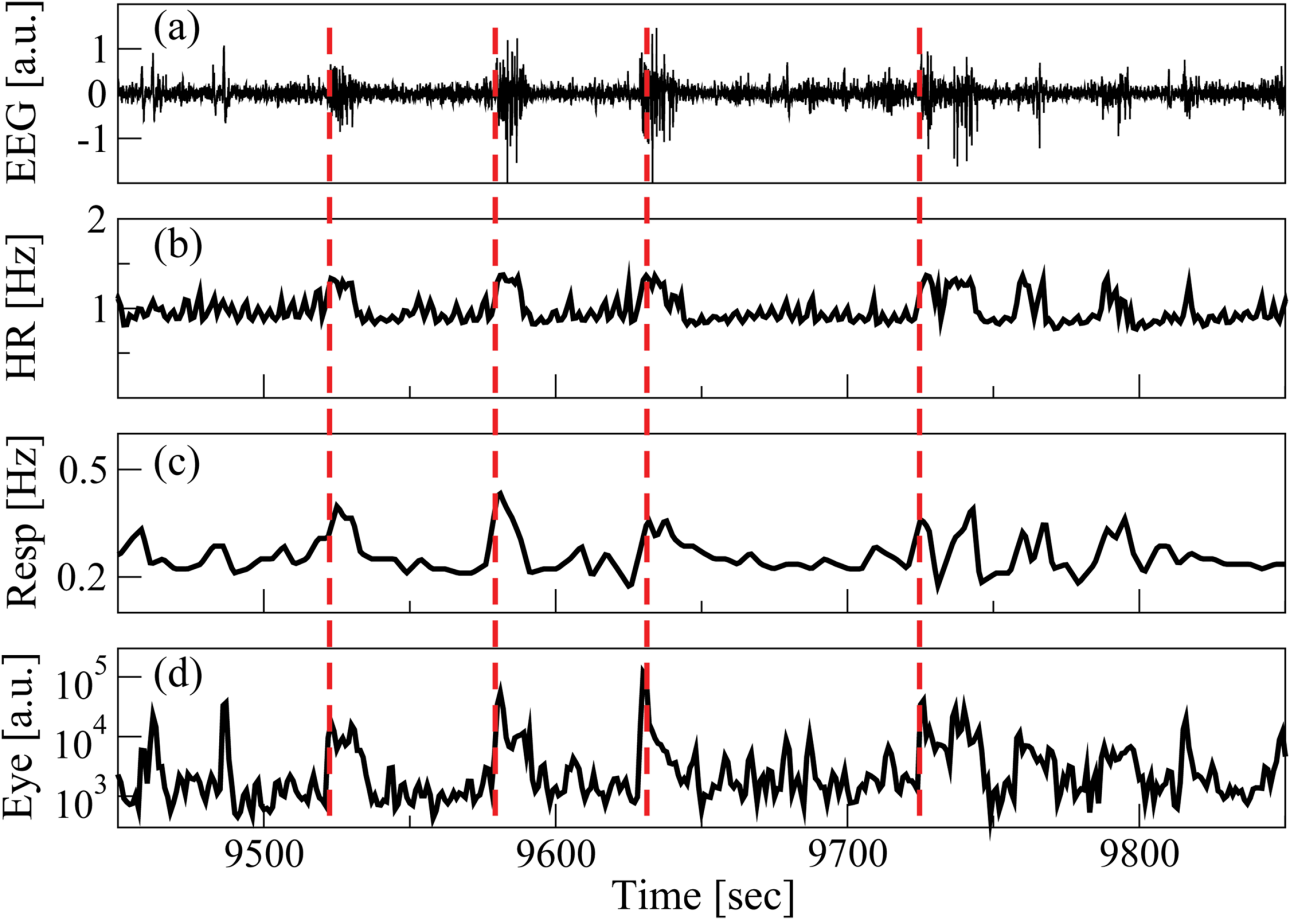
Coordinated bursting activities in the output signals from diverse physiological organ systems. Segments of synchronously recorded physiological signals including (a) brain EEG signal (EEG), (b) heart rate (HR), (c) respiratory rate (Resp) (d) eye movement recording (Eye). Coordinated bursting activities with certain time delay are consistently observed across the output signals of organ systems. Red dashed lines highlight a train of four significant bursts. These bursts transcend all systems and indicate networked communications among the systems.

Due to the levels of complexity inherent to physiologic systems and their dynamical interactions as a network (Section Introduction), currently there is no general theoretical framework and computational intstrumentarium able to simultaneously quantify coupling among diverse systems. Thus, we develop a new physics approach that is general enough to identify and quantify pair-wise coupling and network interactions of diverse dynamical systems. This approach is inspired by observations of coordinated bursting activity in the output dynamics of such systems (Fig 1).

**Fig 2:**
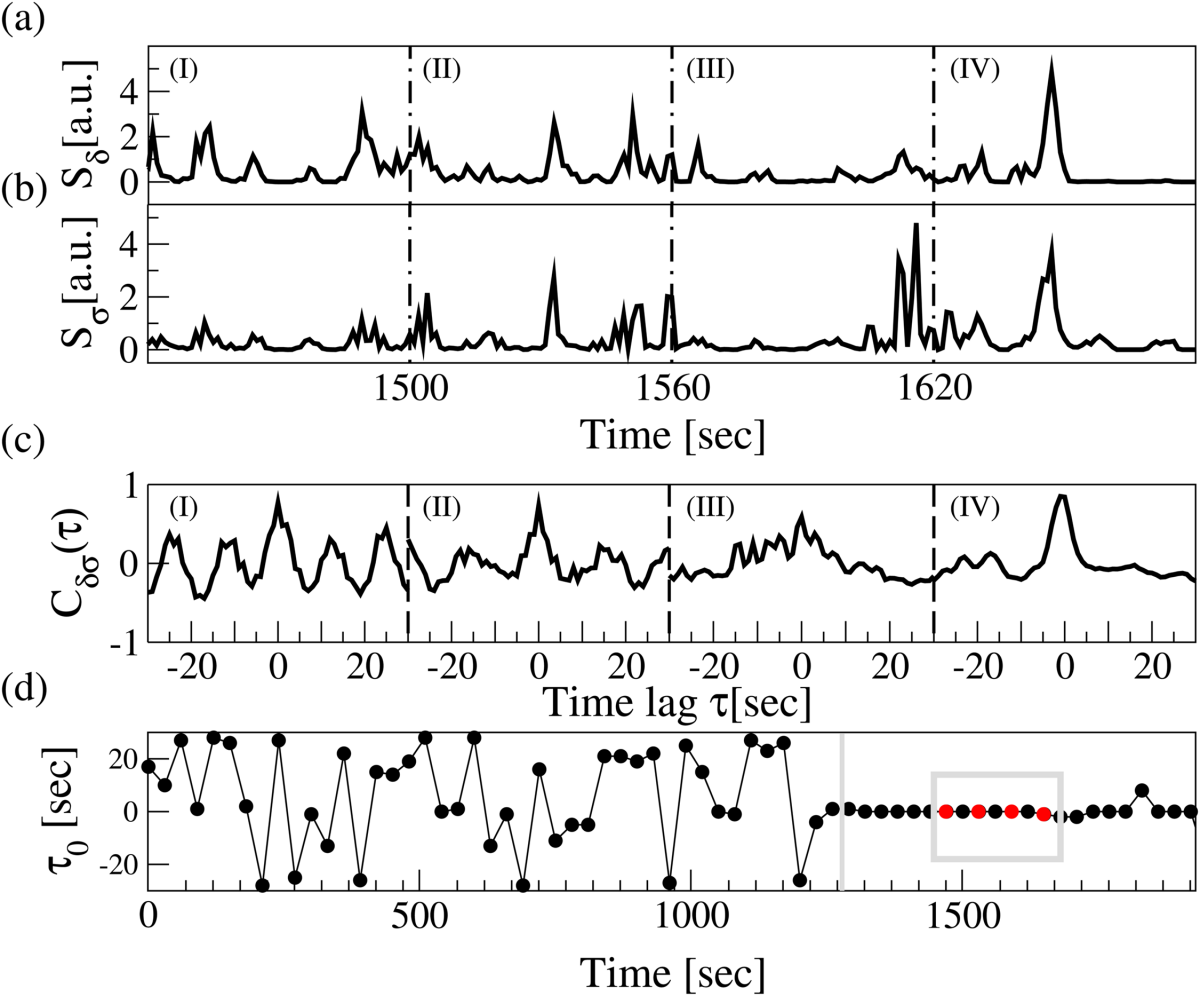
Schematic presentation of the TDS method. Segments of brain EEG power spectrum *S*(*f*) for the (a) *δ* band and (b) *σ* band shown in 60 sec time windows (I), (II), (III) and (IV). Synchronous bursts in *S*_*δ*_ and *S*_*σ*_ lead to pronounced cross-correlation (shown in (c)) within each time window in (a) and (b), and to a stable time delay characterized by segments of constant *τ*_0_ as shown in (d)—e.g., four red dots highlighted by a grey box in panel (d) represent the time delay in the cross-correlation function peaks for each of the 4 time windows in (c). Note the transition from strongly fluctuating behavior in *τ*_0_ to a stable time delay regime at the transition from deep sleep to light sleep at ∼1200 sec in panel (d). The TDS analysis (See Section Method) is performed on overlapping moving windows with a step of 30 sec. Long periods of constant time delay *τ* indicate strong TDS coupling represented by strong links in the network of physiologic interactions. The TDS approach is general and can identify and quantify interactions between systems with diverse dynamics and characteristic time scales.

Integrated physiologic systems are coupled by non-linear feedback and/or feed forward loops with a broad range of time delays [31]. To probe the network of physiologic coupling we introduced a novel concept, Time Delay Stability (TDS), and we recently developed a new TDS method [1, 2, 10] to study the time delay with which bursts of activation in the output dynamics of a given system are consistently followed by corresponding bursts in the signal output of other systems (Fig 1)—periods of TDS with constant time delay between bursts in the activation of two systems indicate stable interactions, and correspondingly stronger coupling between systems results in longer periods of TDS (Fig 2). Thus, the links strength in the physiologic networks we investigate is determined by the percentage of the time when TDS is observed: higher percentage of TDS (%TDS) corresponds to stronger links.

To quantify the interaction between distinct physiologic systems A and B, we consider their output signals {*a*} and {*b*} each of length *N*. We divide both signals {*a*} and {*b*} into *N*_*L*_ overlapping segments *ν* of equal length *L* = 60*s*. We choose an overlap of *L*/2 = 30*s*, which corresponds to the time resolution of conventional sleep-stage-scoring epochs, and thus *N*_*L*_ = [2*N*/*L*] − 1. Before the analysis, the signals in each segment *ν* is normalized separately to zero mean and unit standard deviation, in order to remove constant trends in the data and to obtain dimensionless signals. This normalization procedure assures that the estimate coupling between the signals {*a*} and {*b*} is not affected by their relative amplitudes. Next, we calculate $C_{ab}^{\nu }\left(\tau \right)=\frac{1}{L}\sum_{i=1}^{L}a_{i+(\nu -1)L/2}^{\nu }b_{i+(\nu -1)L/2+\tau }^{\nu }$, which is the cross-correlation function within each segment *ν* ∈ [1, *N*_*L*_] by applying periodic boundary conditions. For each segment *ν*, we define the time delay $\tau_{0}^{\nu }$ to correspond to the maximum in the absolute value of the cross-correlation function $C_{ab}^{\nu }\left(\tau \right)$ in the is segment (Fig 2(c)).

Time periods of stable interrelation between two signals are represented by segments of approximately constant *τ*_0_ in the newly defined series of time delays, $\left\{\tau_{0}^{\nu }\right\}{|}_{\nu \in [1,N_{L}]}$. In contrast, absence of stable coupling between the signals corresponds to large fluctuations in *τ*_0_. We identify two systems as linked if their corresponding signals exhibit a time delay that does not change by more that ±1 for several consecutive segments *ν*. We track the values of *τ*_0_ along the series $\left\{\tau_{0}^{\nu }\right\}$: when for at least four out of five consecutive segments *ν* (corresponding to a window of 5 × 30*s*) the time delay remains in the interval [*τ*_0_ − 1, *τ*_0_ + 1], these segments are labelled as stable (Fig 2(d)). This procedure is repeated for a sliding window with a step size one along the entire series $\left\{\tau_{0}^{\nu }\right\}$. The % TDS is finally calculated as the fraction of stable points in the time series $\left\{\tau_{0}^{\nu }\right\}$.

The TDS method is robust and can track in fine temporal detail (1min windows) how the network of interaction among organ systems change in time with transitions across physiologic states (Fig 2). The TDS method provides a general framework that can be applied to diverse systems with very different types of output dynamics (oscillatory, stochastic or mixed), and does not have the limitations of cross-correlation method where results are affected by the auto-correlations of the analyzed signals, or the limitations of synchronization method applicable only to systems with oscillatory dynamics. Further, the method involves a detrending procedure to address problems with non-stationarity of physiologic signals. By probing the coupling through the time delay in the bursting activity of the output signals, the TDS approach can adequately quantify coordination between physiologic systems even when systems communicate through multiple independent forms of coupling that switch on/off and can simultaneously coexist [10, 32].

Employing the novel concept of Time Delay Stability, we are creating the first tools that enable us to rigorously explore the way in which physiological systems integrate as a network to produce distinct physiologic functions.

### Graphical visualization of physiologic networks

To dissect information hidden in the large TDS matrix where each matrix element represents the pair-wise coupling between organ systems as measured by the %TDS, we develop a graphical representation framework to map complex networked physiologic interactions onto two-dimensional graphical objects encoded with information on the coupling strength between the systems calculated by the TDS method.

This graphical approach is essential to identify universal patterns in the network structure and to track the transition in network characteristics across different physiologic states. This enable us to investigate simultaneous interactions among diverse organ systems mediated through various frequency bands and to search for association between physiologic network topology and physiologic function. The approach lays the foundation to build first maps of dynamic organ interactions in the human organism.

#### Brain-Brain Networks

**Fig 3:**
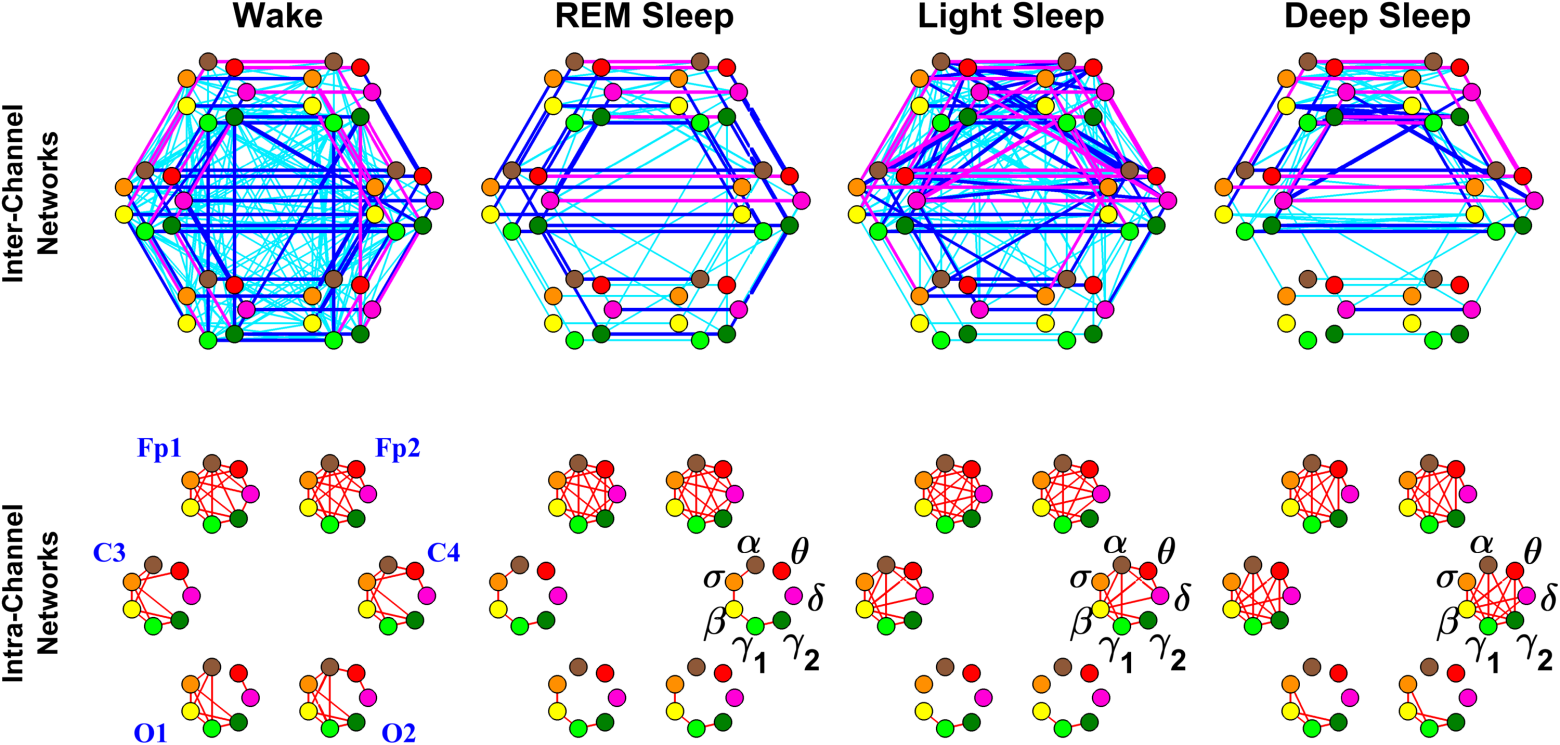
Inter- and intra-channel brain networks during different physiologic states. Network nodes with different colors represent seven different frequency bands (*δ*, *θ*, *α*, *σ*, *β*, *γ*_1_, *γ*_2_) derived from EEG signals (see Section Data), and each set of seven nodes ordered as a heptagon forms a vertex on the hexagon representing six EEG channels from particular brain locations: 2 Frontal areas (Fp1 and Fp2), 2 Central areas (C3 and C4) and 2 Occipital areas (O1 and O2). Coupling strength between frequency bands of signals from different EEG channels (i. e., inter-channel networks) is quantified as the fraction of time (out of the total duration of a given sleep stage throughout the night) when TDS is observed (upper panel). While during quiet W and LS the network of inter-channel brain interactions exhibit high connectivity and strong links between frequency bands of different EEG channels, the networks during REM and DS are more sparse with weaker links. Links between frequency bands of the same EEG channel (i.e., intra-channel networks) are shown in the lower panel. Note that the characteristics of the intra-channel networks for the Frontal areas do not exhibit sleep-stage dependence. In contrast, Central areas exhibit pronounced sleep-stage stratification in network structure, while intra-channel networks in the Occipital areas are more sparse for all sleep stages. Links between two nodes represent the group averaged coupling strength between frequency bands over all subjects. Inter-channel links strength is divided into three categories: very strong links with %TDS≥80% (thick magenta lines); strong links with 65%≤%TDS<80% (thick blue lines); and intermediately strong links with 45%≤%TDS<65% (thin cyan lines). For the intra-channel networks, only links with %TDS≥45% are shown.

Networked interactions between different brain areas mediated through various frequency bands are mapped onto a network that consists of six heptagons each representing individual EEG channels (Fp1, Fp2, C3, C4, O1 and O2) and located at the six corresponding brain areas (2 Frontal, 2 Central and 2 Occipital areas), forming a hexagon (Fig 3).

Each node in the heptagons is assigned a color and represents a specific physiologically-relevant frequency band identified in the EEG spectral power derived from the EEG signal recorded at the corresponding brain EEG-channel location (represented by the heptagon).

These networks are undirected graphs where inter-channel links strength is divided into three categories: very strong links with %TDS≥80% (thick magenta lines); strong links with 65%≤%TDS<80% (thick blue lines); and intermediately strong links with 45%≤%TDS<65% (thin cyan lines).

**Fig 4:**
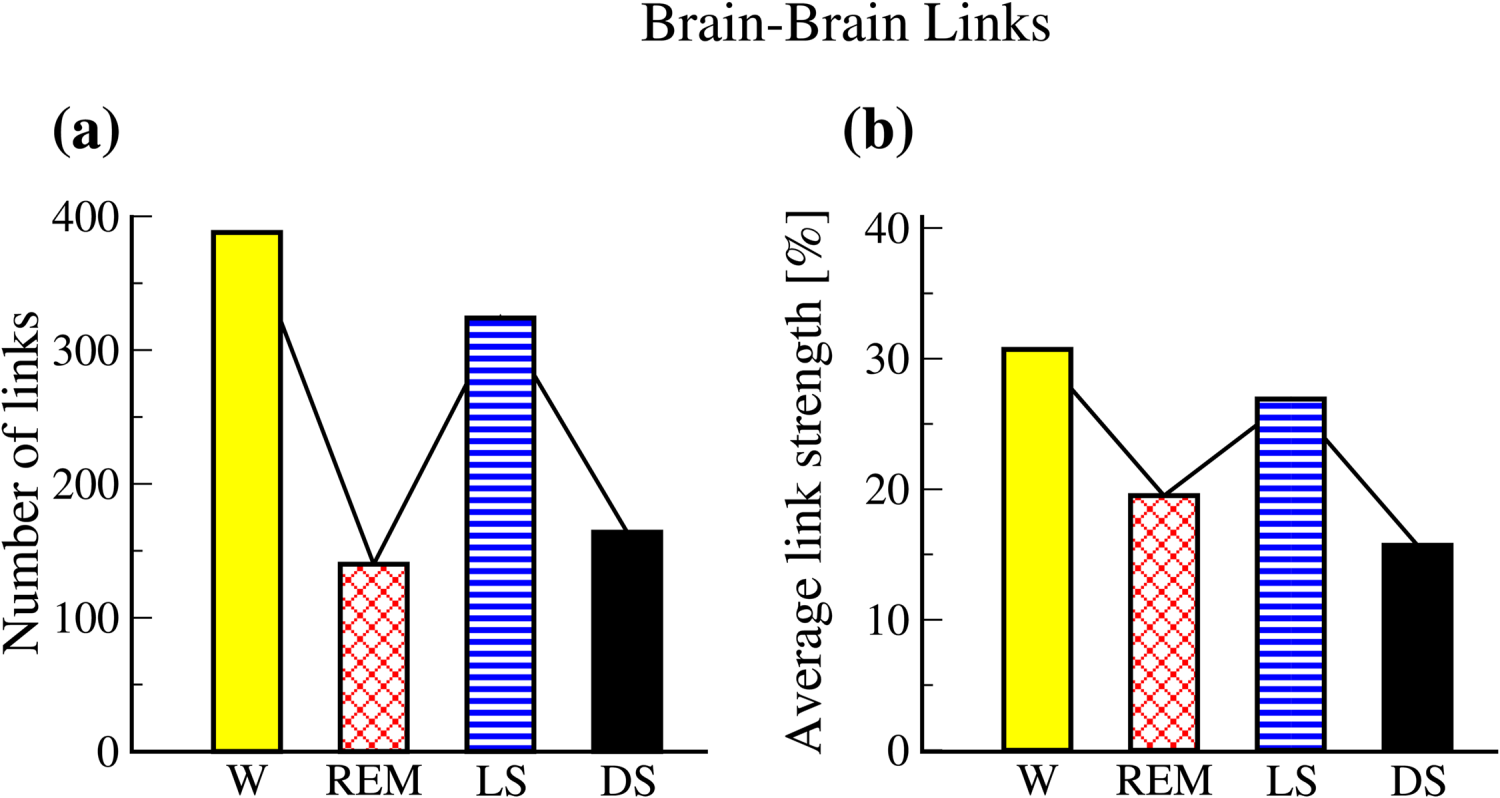
Network characteristics of brain-brain interactions of the inter-channel sub-networks. Pronounced sleep-stage stratification pattern is observed in both (a) number of links and (b) group averaged links strength. Notably, there is a significant difference between LS and DS, as well as between W and REM. This is in contrast to traditional sleep-stage classification of physiologic dynamics of individual systems, where DS and LS are considered similar states grouped as Non-REM state, that is separate from REM and W.

**Fig 5:**
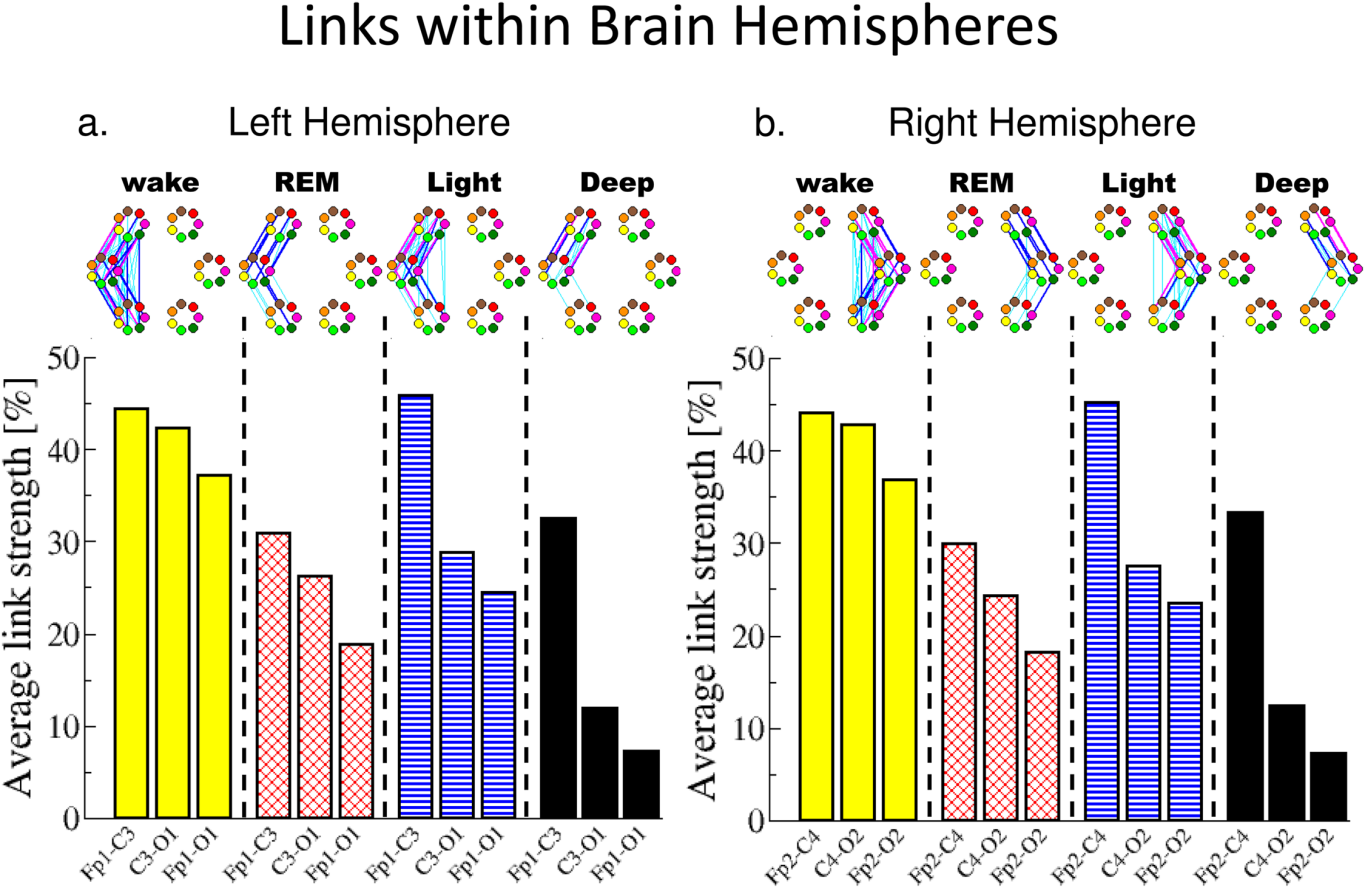
Network structure and links strength of inter-channel brain-brain interactions within each brain hemisphere during different sleep stages. Inter-channel brain links are separately grouped by physiologic state and specific hemisphere. Each group contains three sets of links: Frontal-Central, Central-Occipital and Frontal-Occipital. Network nodes with different colors represent seven different frequency bands (*δ*, *θ*, *α*, *σ*, *β*, *γ*_1_, *γ*_2_), as shown in Fig 3. Inter-channel links strength is divided into three categories: very strong links with %TDS≥80% (thick magenta lines); strong links with 65%≤%TDS<80% (thick blue lines); and intermediately strong links with 45%≤%TDS<65% (thin cyan lines). A very different network structure is associated with each sleep stage, and a hierarchical reorganization involving specific building blocks is observed with transitions across sleep stages (top panels in (a) and (b)). Sleep-stage specific network structure is coupled with pronounced rank order in the links strength across brain areas: strongest links between Frontal and Central areas; intermediate links between Central and Occipital areas and weaker links between Frontal and Occipital areas. Notably, this rank order exhibits pronounced stratification pattern across sleep stages. Both network structure and links strength show a remarkable symmetry between the left and right hemisphere.

**Fig 6:**
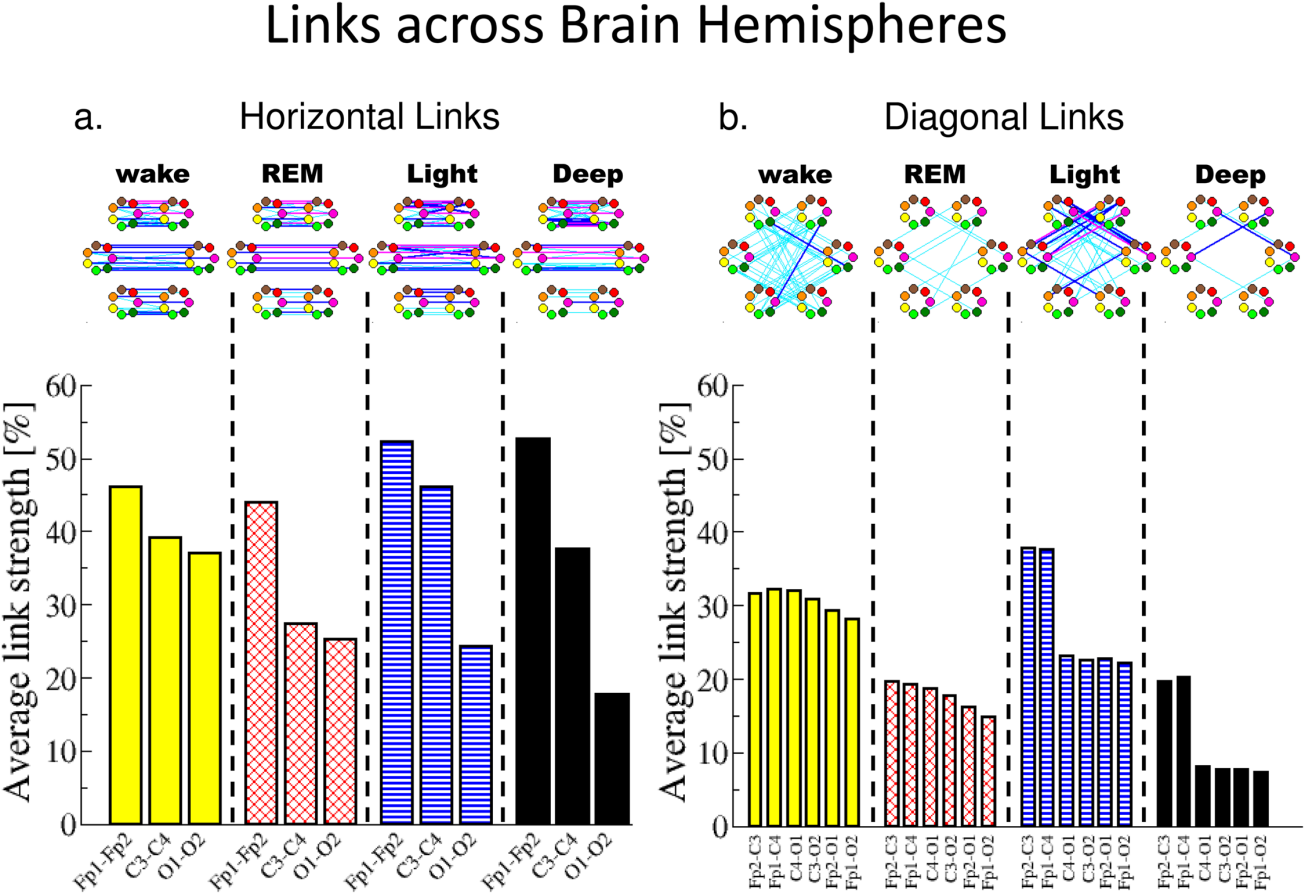
Network structure and links strength of inter-channel brain-brain interactions across brain hemispheres during different sleep stages. Inter-channel brain links are grouped by physiologic state and are separately plotted for: (a) the horizontal links across the left and right brain hemisphere including three subsets: Frontal-Frontal, Central-Central and Occipital-Occipital links; and (b) the diagonal links including six sets of cross-hemisphere links: 2 Frontal-Central, 2 Central-Occipital and 2 Frontal-Occipital links. Network nodes with different colors represent seven different frequency bands (*δ*, *θ*, *α*, *σ*, *β*, *γ*_1_, *γ*_2_), as shown in Fig 3. Inter-channel links strength is divided into three categories: very strong links with %TDS≥80% (thick magenta lines); strong links with 65%≤%TDS<80% (thick blue lines); and intermediately strong links with 45%≤%TDS<65% (thin cyan lines). A distinct network structure is associated with each sleep stage ((a) and (b), top panels). Further, a consistent rank order in the group averaged links strength and a pronounced sleep-stage stratification pattern is observed for all cross-hemisphere inter-channel networks (similar to the one observed for the networks within each hemisphere shown in Fig 5), indicating a basic rule of brain-brain network dynamics.

To further dissect the brain-brain interactions, the entire networks are presented separately as inter-channel networks, intra-channel networks (Figs 3 and 4), networks within each hemispheres (Fig 5) and networks across hemispheres (Fig 6).

#### Brain-Organ Networks

We develop a radar-chart representation to map the brain-organ interactions from across different brain areas and mediated through different frequency bands onto a single network.

Each brain-organ network is represented by (i) six heptagons, one for each of the six brain areas (two Frontal, two Central and two Occipital areas) corresponding to the locations of the EEG channels (Fp1, Fp2, C3, C4, O1 and O2), and (ii) a centered hexagon representing the organ. Each node in the heptagons is assigned a color and represents a specific physiologically-relevant frequency band identified in the EEG spectral power derived from the EEG signal recorded at the corresponding brain EEG-channel location (represented by the heptagon). Brain heptagons are connected to the organ hexagon by links where coupling strength is represented by the line thickness, and where the color of each link corresponds to the color of the connected brain heptagon node (EEG frequency band). Only links above a statistically significant threshold are shown.

**Fig 7:**
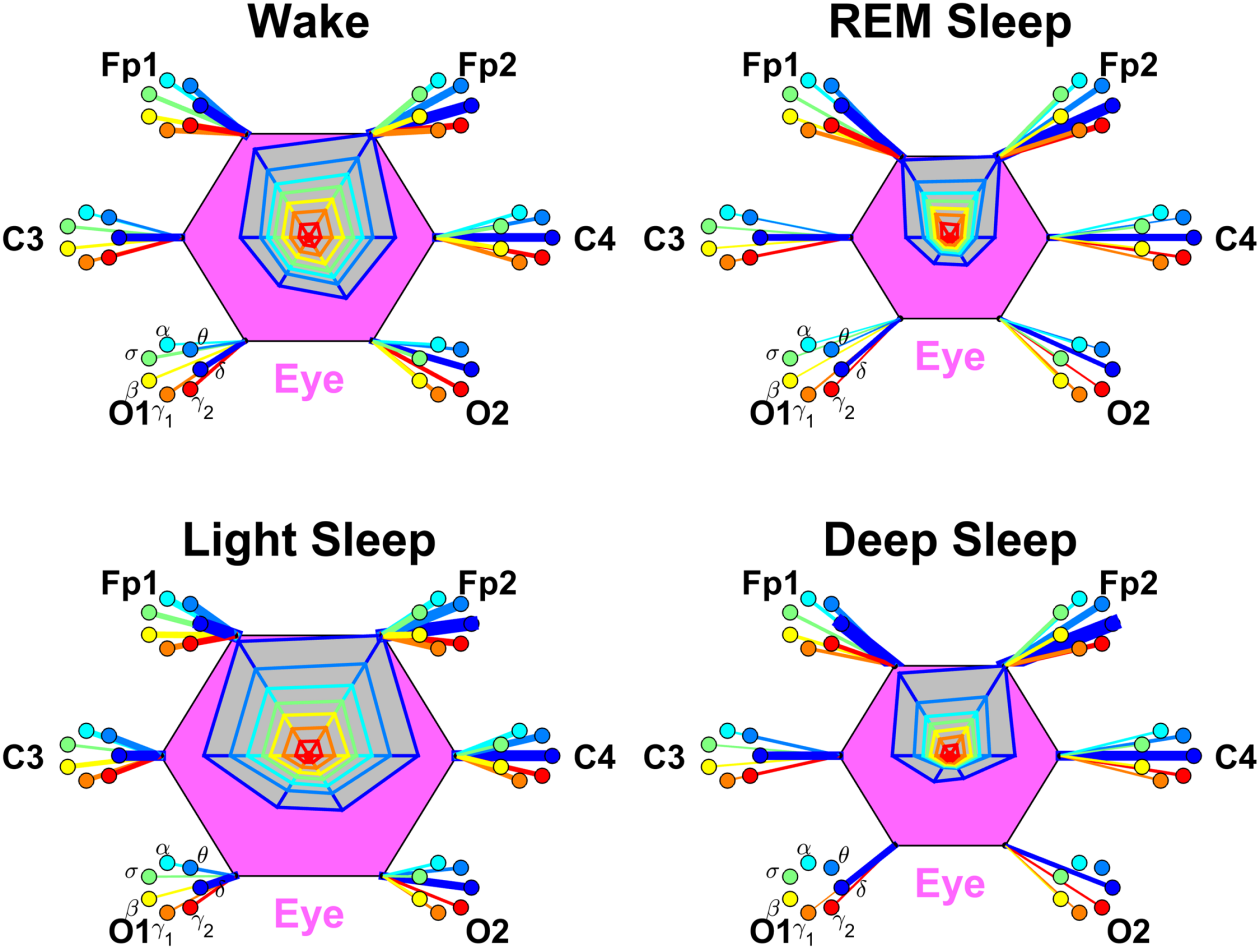
Networks of brain-eye interactions during different physiologic states. Brain areas are represented by Frontal (Fp1 and Fp2), Central (C3 and C4) and Occipital (O1 and O2) EEG channels. Network nodes with different colors represent seven frequency bands (*δ*, *θ*, *α*, *σ*, *β*, *γ*_1_, *γ*_2_) in the spectral power of each EEG channel. Network links between the eye (pink hexagon) and EEG frequency nodes at different locations are determined based on the TDS measure (See Section Methods), and links strength is illustrated by the line thickness. Shown are all links with strength ≥5%TDS. Radar-charts centered in each hexagon represent the relative contribution of brain control from different brain areas to the strength of network links during different sleep stages. The length of each segment along each radius in the radar-charts represents TDS coupling strength between the eye and specific each frequency band at each EEG channel location. These segments are shown in the same color as the corresponding EEG frequency nodes. The brain-eye network interactions are mediated mainly through low-frequency *δ* bands (thick blue links). Dominant links to the Frontal brain areas are observed for all sleep stages and indicate strong spatial asymmetry in brain-eye network interactions. A network re-organization is observed with transition from one sleep stage to another with a pronounced stratification pattern in the overall strength of network links—higher strength in W and LS (larger hexagons) and weaker links during REM and DS (smaller hexagons).

A radar-chart centered in the organ hexagon represents the relative contribution of brain control from different brain areas to the strength of brain-organ network links (e.g., as shown in Fig 7). The length of each segment along each radius in the radar-charts represents TDS coupling strength between the organ and a specific frequency band at each EEG channel location. These segments are shown in the same color as the corresponding heptagon nodes (EEG frequency bands).

Compared to traditional data representation such as histograms and correlation matrices, our visualization framework is complimentary and physiologically motivated. It is useful for studying spatial distribution of brain control and its change in time with transitions across different physiologic states. By associating links strength with line thickness and assigning colors to frequency bands, we are able to identify dominant interaction and the corresponding frequencies that mediate different brain-organ network interactions.

To obtain an integrated description of how the body’s multifarious systems coordinate with one another to produce distinct physiologic functions, we map the interactions of all organs with the brain onto a single network with 5 organ nodes and 42 brain nodes corresponding to 7 different frequency bands (heptagons) and 6 different brain locations (Section Results: Brain-Organ Networks). We plot all brain-organ interactions (network links) with different colors for different frequency bands and with different line width for different coupling strength. We also assign colors to the organ nodes to indicate the dominant frequency bands through which the brain-organ interactions are mediated, and we scale the size of each organ node to the overall strength of TDS coupling between the organ and the brain. This approach enable us to identify and display the reorganization of the global network configuration across distinct physiologic states as well as the frequency bands through which the brain-organ interactions are mediated for different organ systems.

#### Organ-Organ Networks

We develop maps of organ-organ communication that encode the %TDS coupling strength with different width and darkness of the links.

The overall brain control of a given organ (i. e., total TDS coupling strength from all brain channels and different frequencies) is represented by the size of organ nodes in the organ-organ network.

To better understand how organ-organ interactions are influenced by the relative contribution to links strength from different frequency bands and different brain areas (as represented by the shape of radar-chart in the organ hexagon), we scale all organ hexagons to standard size and assign colors to organ nodes and organ hexagons according to the dominant brain frequency bands mediating the interaction (Section Results: Organ-Organ Networks).

These maps of organ-organ networks integrate brain control of each organ system with the organ-organ network interactions, and provide a holistic perspective of the entire physiologic network of key organ systems in the human organism and how it changes during different physiologic states.

## Results

The brain is the major center of neurophysiologic control of key organ systems. Brain control mechanisms of organ systems change with different physiologic states and are influenced by complex communications across brain areas through various frequency bands. Thus, as a first step, we investigate networks of brain-brain interactions representing communications between different brain areas. To probe the dynamics of brain-brain network interactions and their time evolution across distinct physiologic states, we apply our TDS approach (Section Methods) to EEG measurements of cortical activation at different locations: frontal (Fp1 and Fp2), central (C3 and C4) and occipital (O1 and O2).

Further, it is important to know how brain areas communicate through various frequency bands, since brain activity during different physiologic states is associated with specific brain waves and dominant frequencies—e.g., *α*–wave dis-synchronous cortical activation during quiet wake (W) and REM, *θ*–wave during light sleep (LS) and global synchronous *δ*–wave activation during deep sleep (DS). Therefore, we build a dynamical network where nodes represent 7 physiologically-relevant frequency bands (*δ*, *θ*, *α*, *σ*, *β*, *γ*_1_, *γ*_2_) at 6 different EEG channel locations representing distinct cortical areas (See Section Data).

Since neural control of organ systems changes under different physiologic states [24, 25, 28], we hypothesize that specific network topology and dynamics of interactions underlie the control mechanisms across different physiologic states. To this end, we investigate whether and how brain-brain network interactions evolve across different sleep stages which are well-defined physiologic states. Specifically, we aim to identify and quantify interrelations between bursting activities in the spectral power of different frequency bands across brain areas and focus on couplings that remain stable over long time scales (>2.5 min).

### Brain-Brain Networks

We systematically examine the Time Delay Stability between all possible pairs of brain-brain network nodes (overall 861 possible links). We discover that different sleep stages are characterized by markedly different network structure (Fig 3), indicating a clear association between network topology and physiologic function. Further, we find that general network connectivity dramatically changes across sleep stages—fewer links during DS and REM, and much higher connectivity during LS and W, forming a clear sleep-stage stratification pattern (Fig 4a). Remarkably, this stratification pattern is also preserved when we consider the average link strength of the brain-brain network (Fig 4b).

#### Inter-channel brain-brain networks

We uncover that different dominant structures (“building blocks”) underlie general network connectivity and link strength during different sleep stages. Specifically during DS, we find that the network is characterized by strong Frontal-Frontal (Fp1-Fp2) and Central-Central (C3–C4) links. In contrast, Occipital-Occipital (O1–O2) links are less prominent and weaker in strength. In addition, same hemisphere Frontal-Central interactions (Fp1–C3 and Fp2–C4) are more dominant, with higher connectivity and stronger links, compared to Central-Occipital (C3–O1 and C4–O2) interactions. Further, same-hemisphere Frontal-Occipital (Fp1–O1 and Fp2–O2) interactions and cross-hemisphere interactions (diagonal links) are not pronounced during DS (Fig 3, top panel, where interactions with link strength >45% TDS are shown).

Notably, the majority of brain-brain interactions during DS are parallel links between same frequency bands in the Frontal, Central and Occipital locations, while inter-channel interactions across different frequency bands are much weaker and mainly present in the Frontal area. These complex brain-brain inter-channel interactions topologically form a network structure similar to an upper half of a hexagon. This half-hexagon structure is typical for all DS episodes throughout the night and remains present as a building block across all sleep stages (Fig 3, top panel).

Expanding our analysis to REM sleep episodes, we find a similar structure of inter-channel interactions across various frequency bands as observed during DS episodes, however, with more pronounced Occipital-Occipital (O1–O2) and same-hemisphere Central-Occipital (C3–O1 and C4–O2) interactions with higher link strength. Similar to DS, all inter-channel interactions during REM are characterized by dominant interactions between the same frequency bands (parallel links), while links across different frequency bands are much weaker and mainly located in the Frontal area. The increased involvement of Occipital-Occipital and Central-Occipital interactions during REM lead to a network structure that extends the half-hexagon topology (basic building block) observed in DS to a full hexagon configuration. In addition to this hexagonal topology, the inter-channel network of brain interactions during REM is characterized by higher number of weak cross-hemisphere links that are not present in DS (Fig 3, top panel).

Investigating brain-brain interactions during LS, we find that the typical network structure characterized by full-hexagon topology observed for REM is reinforced by stronger links. Moreover, the transition to LS is characterized by a dramatic increase in cross-hemisphere connectivity mediated through much stronger diagonal links, and by the emergence of same-hemisphere Frontal-Occipital links of intermediate strength that are absent in REM and DS. Further, we note that the brain network dynamics during LS are characterized by a significant increase in interactions across different frequency bands not only in the Frontal area (Fp1-Fp2) as observed in REM and DS, but also in the Central area (C3–C4) as well as Frontal-Central interactions (Fp1–C3, Fp2–C4, Fp1–C4 and Fp2–C3). In contrast, interactions across different frequency bands are not observed in the Occipital area (O1–O2) or in Central-Occipital interactions (C3–O1, C4–O2, C4–O1 and C3–O2). Thus our analyses indicate that on top of the typical for REM hexagon topology, the brain-brain network during LS is characterized by additional degrees of cross-hemisphere and cross-frequency bands connectivity in the Frontal and Central areas.

During Wake, we find that brain-brain interactions are characterized by a topology similar to the one observed during LS. However, network connectivity during W is reinforced by additional and stronger links in the Occipital area (O1–O2) as well as by same-hemisphere Central-Occipital (C3–O1 and C4–O2) links. Moreover, in contrast to all other sleep stages, same-hemisphere Frontal-Occipital (Fp1–O1 and Fp2–O2) interactions are characterized by strong network links. Notably, inter-channel brain-brain interactions during W involve a high number of cross-hemisphere and cross-frequency links (Fig 3, top panel), leading to a homogenous network with the highest connectivity (Fig 4a) and the highest average link strength (Fig 4b) compared to all other sleep stages.

#### Intra-channel brain-brain networks

The discussed above results from our TDS analyses of Inter-Channel networks provide novel information of brain-brain communications between different brain areas and how these communications are mediated through different frequency bands during different sleep stages. To better understand the nature of brain dynamics at a given brain area, we next investigate intra-channel networks representing the coordination of brain activation across frequency bands at the same location, i. e. same EEG channel. For each physiologic state, we consider six separate networks corresponding to the six EEG channels (Fp1, Fp2, C3, C4, O1 and O2), where the nodes in each network represent brain wave activation in different physiologically relevant frequency bands.

We uncover that within the same physiologic state (sleep stage), different brain areas exhibit different degree of cross-frequency coupling. In general, Frontal areas (Fp1 and Fp2) are characterized by highly connected networks, indicating strong interactions between all frequency bands; lower connectivity for the Central areas (C3 and C4) and lowest connectivity in the Occipital areas (O1 and O2). This general behavior is further modulated by physiologic states (Fig 3, bottom panel).

We find during all four sleep stages, networks representing Frontal channels exhibit a robust pattern of high connectivity, whereas Occipital channels show practically no network connectivity during REM, LS and DS, and low connectivity only during W. In contrast to Frontal and Occipital channels, networks corresponding to Central channels undergo significant transition in network connectivity cross sleep stages—from almost no connectivity during REM, to low connectivity during W, high connectivity during LS and almost fully connect Central channel networks during DS (Fig 3, bottom panel). Remarkably, a left-right brain hemisphere symmetry in the network connectivity configuration is preserved across all sleep stages.

Further, we find a striking resemblance in network structure for the observed intra-channel networks representing LS and DS. In contrast, REM and W are characterized by a very different intra-channel network structure. These empirical observations are consistent with traditional sleep-stage classification based on EEG spectral measures from the Central channels where, in contrast to REM and W, LS and DS are considered as similar physiologic states and are often grouped together as a single Non-REM state.

Notably, we find that while intra-channel networks have very similar structure during LS and DS (Fig 3, bottom panel), the inter-channel networks for LS and DS exhibit very different topology (Fig 3, top panel), indicating that these two aspects of the TDS network analysis derive complementary information about the nature of brain-brain communications during distinct physiologic states. Indeed, we uncover a different pathway (DS→REM→LS→W) for the hierarchical reorganization of inter-channel networks across different sleep stages, where network connectivity and average link strength during DS are similar to REM, while network characteristics during LS are closer to W.

To better understand the hierarchical organization of inter-channel networks representing communications across brain areas mediated through different frequency bands and how these networks evolve with transitions across physiologic states, we next investigate sections of the inter-channel networks with focus on (i) interactions within brain hemisphere and (ii) interactions across brain hemispheres.

Inter-channel brain-brain networks within brain hemispheres. Considering the sub-network of inter-channel brain interactions mediated through different frequency bands within a given brain hemisphere, we find a specific ranking in the average network link strength—highest for the Frontal-Central interactions, lower strength for Central-Occipital interactions and weakest links for the Frontal-Occipital interactions (Fig 5). This ranking in link strength is observed during all sleep stages and indicates a previously unknown general rule that underlies inter-channel brain interactions across physiologic states.

Further, we find that the average strength of all inter-channel links within the same brain hemisphere exhibits a pronounced sleep-stage stratification pattern—highest average link strength (≈40% TDS) during W; lower (≈25% TDS) during REM; higher (≈32% TDS) during LS; and lowest (≈15% TDS) during DS (Fig 5). This stratification pattern is the same as the one we observed for the entire inter-channel brain network for both hemispheres shown in Fig 4. Moreover, each group of Frontal-Central, Central-Occipital and Frontal-Occipital links within the same hemisphere also follows the same sleep-stage stratification pattern—stronger links during W and LS, weaker links during REM and DS—indicating a common basic regulatory mechanism across physiologic states.

However, the relative differences in the group average strength of Frontal-Central, Central-Occipital and Frontal-Occipital links exhibit distinct profiles for each physiologic state, with small relative differences of <10% during W and <15% during REM versus a dramatic decline in the Central-Occipital and Frontal-Occipital links strength compared to the Frontal-Central links of >30% in LS and >50% during DS (Fig 5).

In summary, we uncover two basic rules in link strength organization of brain-brain interactions, represented by (i) the stable ranking order of the average link strength of inter-channel networks within each brain hemisphere observed for each physiologic state and (ii) the robust sleep-stage stratification pattern in average link strength within each brain hemisphere.

Remarkably, we note that these basic rules in links strength are closely associated with a structured reorganization in network topology, where transitions across sleep stages are represented by a hierarchical building process in network structure: (1) starting from a cluster of Frontal-Central parallel links between the same frequency bands during DS, as a first building block; (2) an extension of this building block with a second cluster of Central-Occipital parallel links between the same frequency bands during REM, forming the second building block; (3) reinforced Central-Occipital parallel links in the second building block and an extension of the topology with additional weak Frontal-Occipital links during LS, leading to the third building block; (4) reinforced Frontal-Occipital parallel links between the same frequency bands in the third building block and emergence of a multitude of cross-frequency links between the Frontal-Central, Central-Occipital and Frontal-Occipital areas during W, forming the fourth building block.

Finally, comparing the sub-networks of inter-channel brain interactions representing the left and right brain hemisphere, we find a remarkable left-right hemisphere symmetry in both the organization of average link strength across brain areas and in network topology (Fig 5a and 5b).

Inter-channel brain-brain networks across brain hemispheres. We next consider the sub-network of inter-channel interactions across the left and right brain hemispheres. We divide the links in this sub-network into two groups: (i) horizontal links representing Frontal-Frontal (Fp1-Fp2), Central-Central (C3–C4) and Occipital-Occipital (O1–O2) interactions (Fig 6a), and (ii) diagonal links representing Frontal-Central(Fp1–C4 and Fp2–C3), Central-Occipital (C3–O2 and C4–O1) and Frontal-Occipital (Fp1–O2 and Fp2–O1) interactions across the two hemispheres (Fig 6b).

We find that each physiologic state (sleep stage) is characterized by the same ranking order for the average strength for the Frontal-Frontal, Central-Central and Occipital-Occipital links—highest average link strength in the Frontal area, intermediate link strength in the Central area and the weakest links in the Occipital area (Fig 6a, bottom panel). This ranking order is systematically observed in all sleep stage episodes throughout the night and for all individual subjects in the database, indicating a general rule underlying the mechanism of network communications across the two hemispheres.

Even though we observe the same ranking order in average link strength for all sleep stages, we find that with transition from one state to another, the strength of cross-hemisphere horizontal links in different areas change very differently: while the Frontal-Frontal links remain strong without significant change across all sleep stages, the Occipital-Occipital links gradually decrease with increasing sleep depth from W to REM, to LS and DS with a significant decline of >50%. In contrast, the average strength of Central-Central links exhibit a pronounced sleep-stage stratification pattern with stronger links in W and LS, and weaker links during REM and DS (Fig 6a, bottom panel).

Further, our analyses show that across all sleep stages, Frontal-Frontal interactions always involve both links between the same frequency bands as well as links between different frequency bands. In contrast, Occipital-Occipital interactions during all sleep stages are predominantly mediated through links between the same frequency bands as represented by parallel horizontal links in Fig 6a (top panel). Finally, our analyses indicate that the Frontal-Frontal interactions always involve links between different frequency bands, while Occipital-Occipital interactions practically do not involve such cross-frequency links—a behavior consistent for all sleep stages.

In contrast to this consistent behavior, Central-Central interactions are characterized by the emergence of a significant number of cross-frequency links during W and LS and the absence of such links in REM and DS (Fig 6a, top panel). This sleep-stage stratification in Central-Central cross-frequency links is in addition to the presence of strong Central-Central horizontal parallel links between same frequency bands, thus accounting for the stratification pattern observed in the average links strength for Central-Central interactions(Fig 6a, bottom panel).

We next consider the diagonal links representing Frontal-Central (Fp1–C4 and Fp2–C3), Central-Occipital (C3–O2 and C4–O1) and Frontal-Occipital (Fp1–O2 and Fp2–O1) interactions across the two brain hemispheres (Fig 6b). We find that the group average link strength for all diagonal links across the hemispheres exhibits a sleep-stage stratification pattern with higher average link strength in W and LS, and weaker links during REM and DS (Fig 6b, bottom panel). This pattern is similar to what we find for the average links strength for the same-hemisphere links (Fig 5) and is also consistent with the behavior with the entire network including all possible brain-brain links (Fig 4b).

Notably, we observe the same sleep-stage stratification pattern also in the number of diagonal links across hemispheres with higher number of links during W and LS, lower number of links during REM and much weaker network connectivity during DS (Fig 6b, top panel). Moreover, we find that the structural re-organization of network connectivity across sleep stages follows the same stratification pattern for the sub-network of diagonal links across brain hemispheres (Fig 6b, top panel), for the sub-network of links within a given hemisphere (Fig 5a and 5b, top panels), as well as the entire network of all brain-brain interactions (Fig 4a). Such common stratification pattern indicates a clear association between network topology and the strength of brain-brain interactions that may result from a common underlying mechanism regulating these two aspects of brain network interactions with transitions across sleep stages.

### Brain-Organ Networks

To better understand the neurophysiologic control of key organ systems, we next focus our investigation on identifying and quantifying the networks of interactions between the brain and individual organ systems. Specifically we consider the following key organs: eye, chin, leg, heart and respiratory system.

There are several key questions related to the nature of brain-organ interactions which we systematically investigate for all organ systems: (i) how different areas of the brain as represented by different EEG-channel locations are involved in the communications and control of each organ system, (ii) which brain-wave frequency bands mediate the brain-organ communications, and (iii) how the networks representing brain-organ interactions across brain areas and different brain-wave frequency bands evolve with transitions across physiologic states.

To this end, we apply the TDS method (Section Methods) to identify and quantify dynamical links in the networks of brain-organ interactions, and we develop radar-charts (Section Methods) to graphically represent these complex communications, and how they change with physiologic states. The obtained networks serve as unique physiological maps of brain-organ interactions. These maps are further supported by detailed histograms with quantitative assessment of the strength of brain-organ interactions for specific brain areas and frequency bands across different physiologic states.

#### Brain-eye interactions

We find that the network of brain-eye interactions exhibits a strong spatial asymmetry where network links between brain and the eye are strongest for the Frontal brain areas (Fp1 and Fp2 channels), intermediate for the Central areas (C3 and C4) and very weak for the Occipital areas (O1 and O2). This spatial asymmetry in the average brain-eye link strength is shown by the asymmetric radar chart inside the eye-hexagon and is systematically observed for all sleep stages (Fig 7).

**Fig 8:**
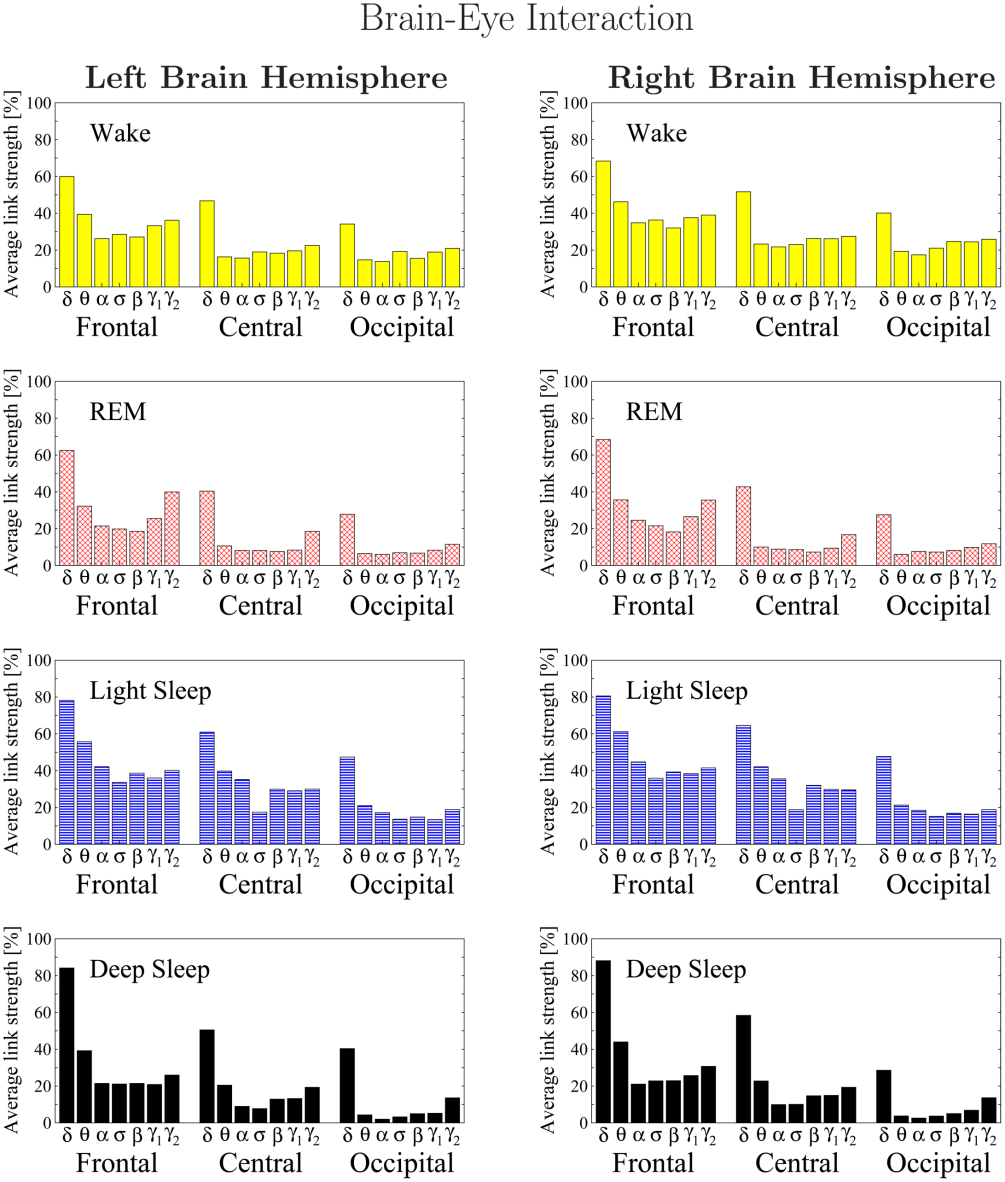
Histograms of links strength in the brain-eye network during different sleep stages. Group averaged links strength are obtained using the TDS measure, where each link represents the interaction of the eye with a given brain area through a specific frequency band. Links are separately grouped by brain areas (Frontal, Central or Occipital) and are arranged in order from low-frequency (*δ* and *θ*) to high-frequency (*γ*_1_ and *γ*_2_) bands. A consistent frequency profile of links strength is observed for brain areas—strongest links mediated through the low-frequency *δ* band, and weak links mediated through the mid-range frequency bands. Links strength between the eye and different brain areas decreases from the Frontal to Central and Occipital areas. The sleep-stage stratification observed for the links strength in the brain-eye radar-charts (Fig 7) is consistent for all frequency bands and brain areas as shown in the histograms. Note the strong symmetry in the links strength distribution between the left and right hemisphere that is present for all sleep stages.

Our analysis of the networks of brain-eye interactions reveals a specific frequency profile in the strength of network links. This frequency profile for the brain-eye links is characterized by highest link strength for the lowest frequency *δ* band, intermediate strength for the low-frequency *θ* and high-frequency *γ*_1_ and *γ*_2_ bands, and lowest strength for the mid-frequency *α*, *σ* and *β* bands. Remarkably, this frequency profile of brain-eye links remains stable for all brain areas (Frontal, Central and Occipital) as shown in Fig 8. Such stable frequency profile together with the observed spatial asymmetry in the average link strength of the brain-eye network (Fig 7) indicate the presence of a robust rank order (Frontal-eye links>Central-eye links>Occipital-eye links) that underlies brain-eye interactions across all frequency bands.

Repeating our network analysis for all 7 frequency bands across different sleep stages, we find that the average link strength for the entire network of brain-eye interactions is highest during W and LS, lower during REM and lowest during DS (Fig 7). Further, the sleep-stage stratification pattern is consistently observed for all three sub-networks representing the Frontal-eye, Central-eye and Occipital-eye links across all frequency bands (Fig 8). Therefore, our observation show that all links in the brain-eye network, regardless of brain areas or frequency bands, are modulated in the same way with transitions across sleep stages, indicating a common mechanism of neural regulation.

Finally, we note that the brain-eye interaction mediated through the lowest-frequency *δ* band plays a special role in the brain-eye network, as the link strength of this interaction remains exceptionally high (≈80%TDS) during LS and DS, which is even higher than the strength of this link (≈60%TDS) during W and REM (Fig 8).

Comparing our results for the brain-eye interactions obtained separately from the left and right brain hemispheres, we find a remarkable left-right symmetry in the strength of all network links across brain areas and frequency bands (Fig 8).

#### Brain-chin interactions

**Fig 9:**
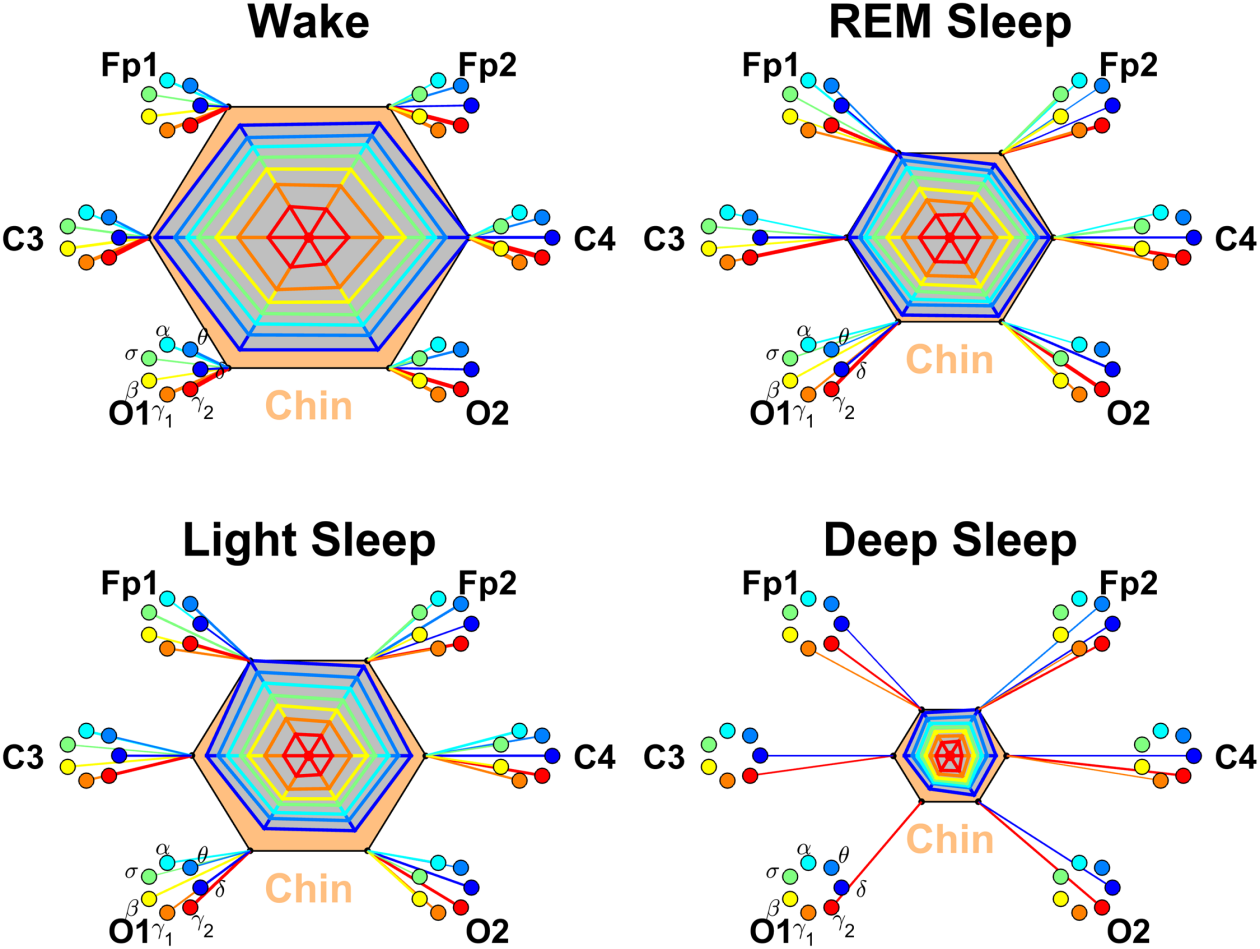
Networks of brain-chin interactions during different physiologic states. Brain areas are represented by Frontal (Fp1 and Fp2), Central (C3 and C4) and Occipital (O1 and O2) EEG channels. Network nodes with different colors represent seven frequency bands (*δ*, *θ*, *α*, *σ*, *β*, *γ*_1_, *γ*_2_) in the spectral power of each EEG channel. Network links between the chin (orange hexagon) and EEG frequency nodes at different locations are determined based on the TDS measure (See Section Methods), and links strength is illustrated by the line thickness. Shown are all links with strength ≥5%TDS. Radar-charts centered in each hexagon represent the relative contribution of brain control from different brain areas to the strength of network links during different sleep stages. The length of each segment along each radius in the radar-charts represents TDS coupling strength between the chin and each frequency band at each EEG channel location. These segments are shown in the same color as the corresponding EEG frequency nodes. The brain-chin network interactions are mediated mainly through high-frequency *γ*_1_ and *γ*_2_ bands (orange and red links). In contrast to the brain-eye network (Fig 7), the brain-chin network is characterized by relatively symmetric links strength to all six brain areas, as shown by the symmetric radar-chart in each hexagon. A pronounced stratification pattern is observed for the overall strength of network links—stronger links during W and LS (larger hexagons) and weaker links during REM and DS (smaller hexagons).

We discover that brain-chin interactions are spatially symmetric, where the strength of network links is similar for the Frontal brain areas (Fp1 and Fp2 channels), the Central areas (C3 and C4) and for the Occipital areas (O1 and O2), as indicated by the symmetric radar chart inside the chin hexagon in Fig 9. This spatial symmetry in the average brain-chin link strength is systematically observed for all sleep stages (Fig 9). This is in contrast to the strong spatial asymmetry we found in brain-eye networks (Fig 7) and suggest a more uniform involvement of brain areas in the neural regulation of chin muscle tone.

**Fig 10:**
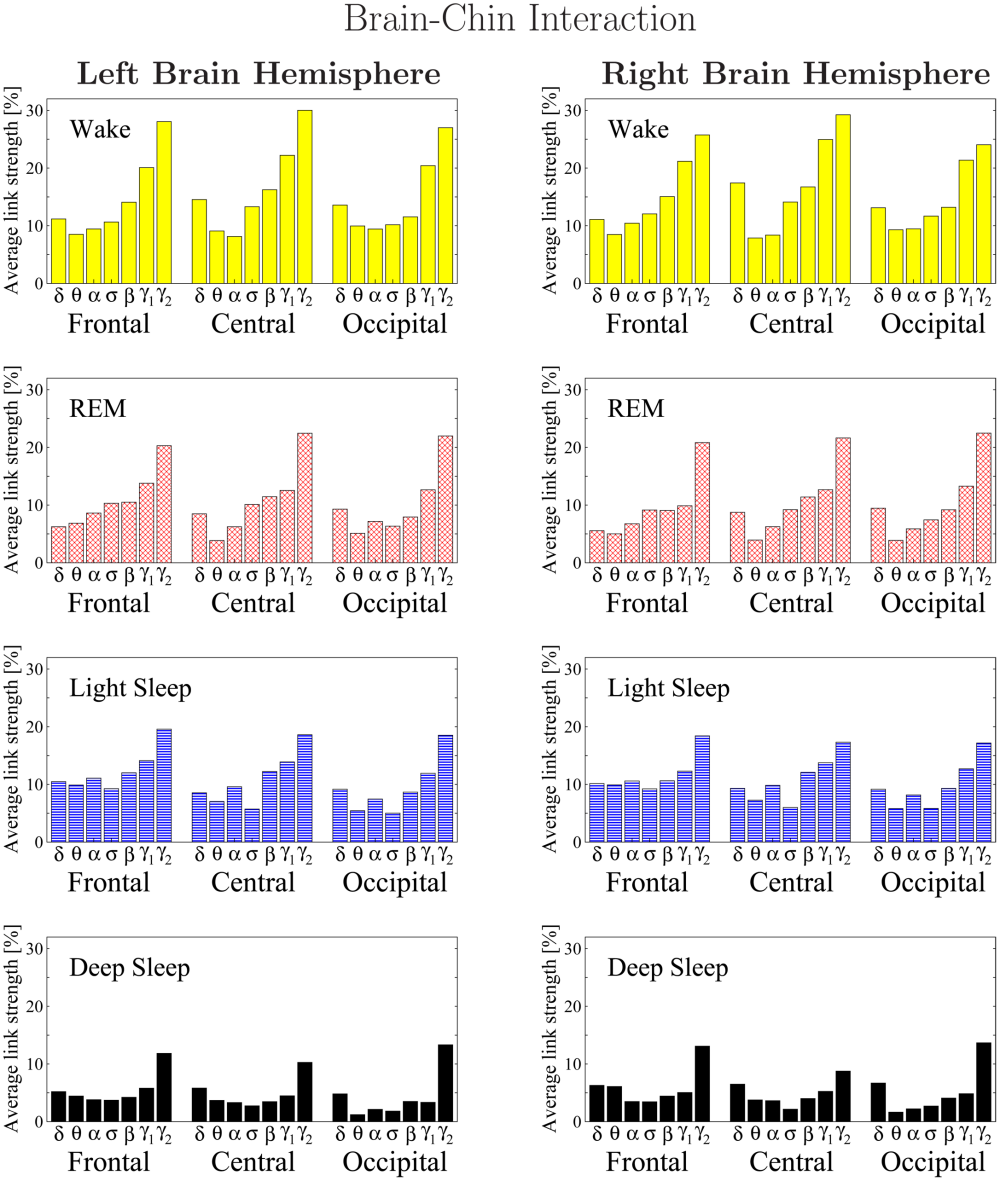
Histograms of links strength in the brain-chin network during different sleep stages. Group averaged links strength are obtained using the TDS measure, where each link represents the interaction of the chin with a given brain area through a specific frequency band. Links are separately grouped by brain areas (Frontal, Central or Occipital), and are arranged in order from low-frequency (*δ* and *θ*) to high-frequency (*γ*_1_ and *γ*_2_) bands. A consistent frequency profile of links strength is observed for all brain areas—strongest links mediated through the highest-frequency *γ*_2_ band, a gradual decrease in link strength for the lower-frequency bands (*γ*_1_, *β*, *σ*, *α*, *θ*), followed by a slight increase in link strength for the lowest-frequency *δ* band. The sleep-stage stratification observed for the links strength in the brain-chin radar-charts (Fig 9) is consistently observed for all frequency bands and brain areas as shown in the histograms—stronger links during W and LS, and weaker links during REM and DS. Note the strong symmetry in the links strength distribution between the left and right hemisphere that is present for all sleep stages.

In addition, our analysis of the networks of brain-chin interactions reveals a very different frequency profile in the strength of network links compared to the brain-eye networks. The frequency profile for the brain-chin links is characterized by strongest links for the highest frequency *γ*_2_ band, a gradual decrease in link strength for the lower frequency bands (*γ*_1_, *β*, *σ*, *α*, *θ*), followed by a slight increase in link strength for the lowest-frequency *δ* band. Remarkably, this frequency profile of brain-chin links remains stable for all brain areas (Frontal, Central and Occipital) as shown in Fig 10. This stable frequency profile for the strength of brain-chin interactions (Fig 10) is very different from the corresponding brain-eye profile (Fig 8), and combined with the symmetric involvement of all brain areas in the brain-chin network (Fig 9) indicates that the underlying dynamics of brain-chin interactions is markedly different from the dynamics that regulate brain-eye communications.

Repeating our brain-chin network analysis for all 7 frequency bands across different sleep stages, we find that the average link strength for the entire network of brain-chin interactions is highest during W and LS, lower during REM and lowest during DS (Fig 9). Further, this sleep-stage stratification pattern is consistently observed for all three sub-networks representing the Frontal-chin, Central-chin and Occipital-chin links across all frequency bands (Fig 10). Thus, our observation show that the strength of all links in the brain-chin network, regardless of brain areas or frequency bands, is modulated in the same way with transitions across sleep stages. This sleep-stage stratification for the brain-chin network is identical to the stratification pattern we found for the link strength in brain-eye network, indicating common aspects in the neural regulation of these two organ systems in relation to sleep-stage transitions.

Comparing our results for the brain-chin interactions obtained separately from the left and right brain hemispheres, we find a remarkable left-right symmetry in the strength of all network links across brain areas and frequency bands (Fig 10). A similar left-right symmetry in link strength was observed for the network of brain-eye interactions (Fig 8).

#### Brain-leg interactions

**Fig 11:**
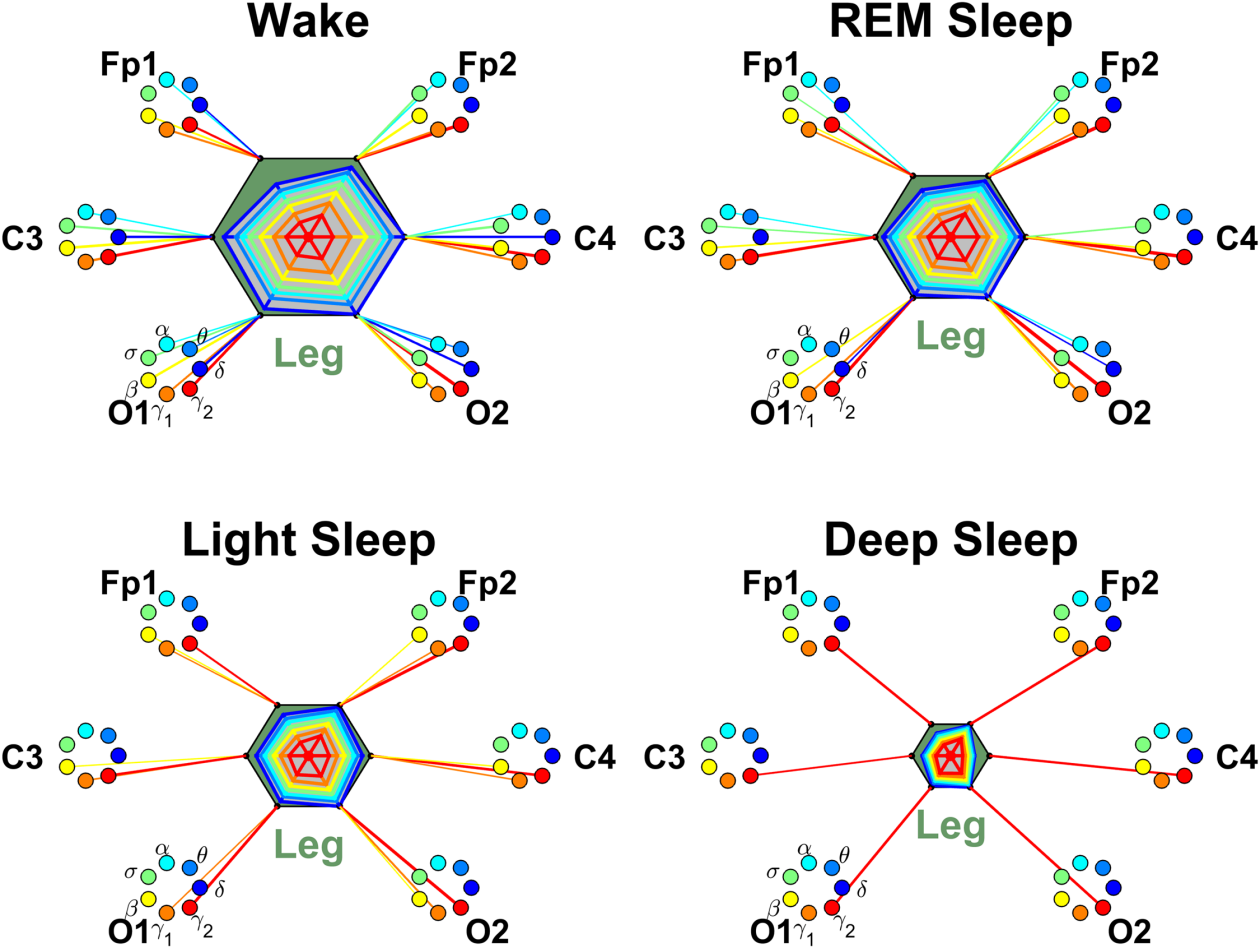
Networks of brain-leg interactions during different physiologic states. Brain areas are represented by Frontal (Fp1 and Fp2), Central (C3 and C4) and Occipital (O1 and O2) EEG channels. Network nodes with different colors represent seven frequency bands (*δ*, *θ*, *α*, *σ*, *β*, *γ*_1_, *γ*_2_) in the spectral power of each EEG channel. Network links between the leg (dark green hexagon) and EEG frequency nodes at different locations are determined based on the TDS measure (See Section Methods), and links strength is illustrated by the line thickness. Shown are links with strength ≥5%TDS. Radar-charts centered in each hexagon represent the relative contribution of brain control from different brain areas to the strength of network links during different sleep stages. The length of each segment along each radius in the radar-charts represents TDS coupling strength between the leg and each frequency band at each EEG channel location. These segments are shown in the same color as the corresponding EEG frequency nodes. The brain-leg network interactions are mediated mainly through high-frequency *γ*_2_ bands (red links). A pronounced stratification pattern is observed for the overall strength of network links—stronger links during W (larger hexagon), intermediate during REM and LS (smaller hexagon) and weaker links during DS. Since the same scaling factor is used to obtain the radar-charts for brain-leg network as for the brain-chin network (Fig 9), smaller radar-charts (smaller hexagons) indicate weaker interactions in the brain-leg network compared to the brain-chin network.

For the network of brain-leg interactions we find that for all sleep stages there is a spatially symmetric distribution for the strength of network links, where links to Frontal brain areas (Fp1 and Fp2 channels), Central areas (C3 and C4) and Occipital areas (O1 and O2) have similar strength, as indicated by the symmetric radar chart inside the leg hexagon in Fig 11. This spatial symmetry in the average brain-leg link strength is similar to the one we observed for the brain-chin network for all sleep stages (Fig 9).

**Fig 12:**
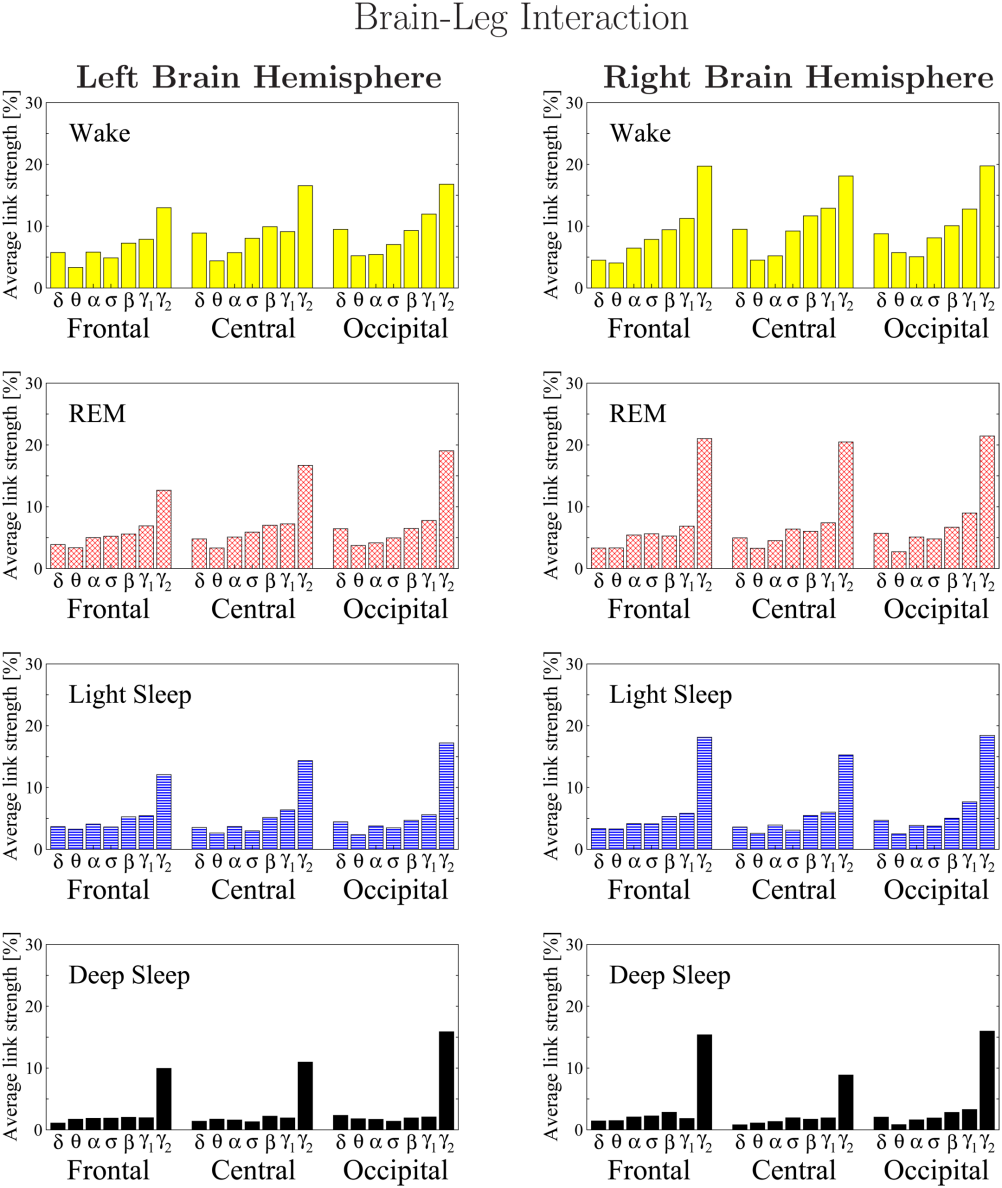
Histograms of links strength in the brain-leg network during different sleep stages. Group averaged links strength are obtained using the TDS measure, where each link represents the interaction of the leg with a given brain area through a specific frequency band. Links are separately grouped by brain areas (Frontal, Central or Occipital), and are arranged in order from low- (*δ* and *θ*) to high-frequency (*γ*_1_ and *γ*_2_) bands. A consistent frequency profile of links strength is observed for all brain areas—strongest links mediated through the highest frequency *γ*_2_ band, followed by a gradual decrease in link strength for the lower-frequency bands (*γ*_1_, *β*, *σ*, *α*, *θ*, *δ*). The sleep-stage stratification observed for the links strength in the brain-leg radar-charts (Fig 11) is consistent for all frequency bands and brain areas as shown in the histograms. Note the strong symmetry in the links strength distribution between the left and right hemisphere that is present for all sleep stages.

Further, we find that the network of brain-leg interactions shares a similar frequency profile in the strength of network links compared to the brain-chin network—strongest links for the highest frequency *γ*_2_ band, followed by a gradual decrease in link strength for the lower frequency bands (*γ*_1_, *β*, *σ*, *α*, *θ*, *δ*). Remarkably, within each physiologic state, the frequency profile of the brain-leg network links remains stable for all brain areas (Frontal, Central and Occipital) as shown in Fig 12.

Extending the brain-leg network analysis for different sleep stages, we find that the average link strength for all 7 frequency bands in the network of brain-leg interactions is highest during W, lower during REM and LS, and lowest during DS (Fig 11). This sleep-stage stratification pattern is consistently observed for all three sub-networks representing the Frontal-leg, Central-leg and Occipital-leg links across all frequency bands (Fig 12), indicating that transitions across sleep stages modulate the strength of all links in the brain-leg network in the same way regardless of brain areas and frequency bands. This sleep-stage stratification in link strength for the brain-leg network is similar to the stratification pattern we found for the brain-eye and brain-chin networks.

Further, our results show a remarkable symmetry between the left and right hemispheres in the strength of all brain-leg network links across brain areas and frequency bands (Fig 12). A similar left-right symmetry in link strength was observed for the network of brain-chin interactions (Fig 10).

The similarity in the spatial distribution and in the frequency profile of links strength we uncover for the brain-leg and brain-chin networks reflect common dynamics underlying neural regulation of muscle tone and a common mechanism of modulation with transition across sleep stages.

#### Brain-heart interactions

**Fig 13:**
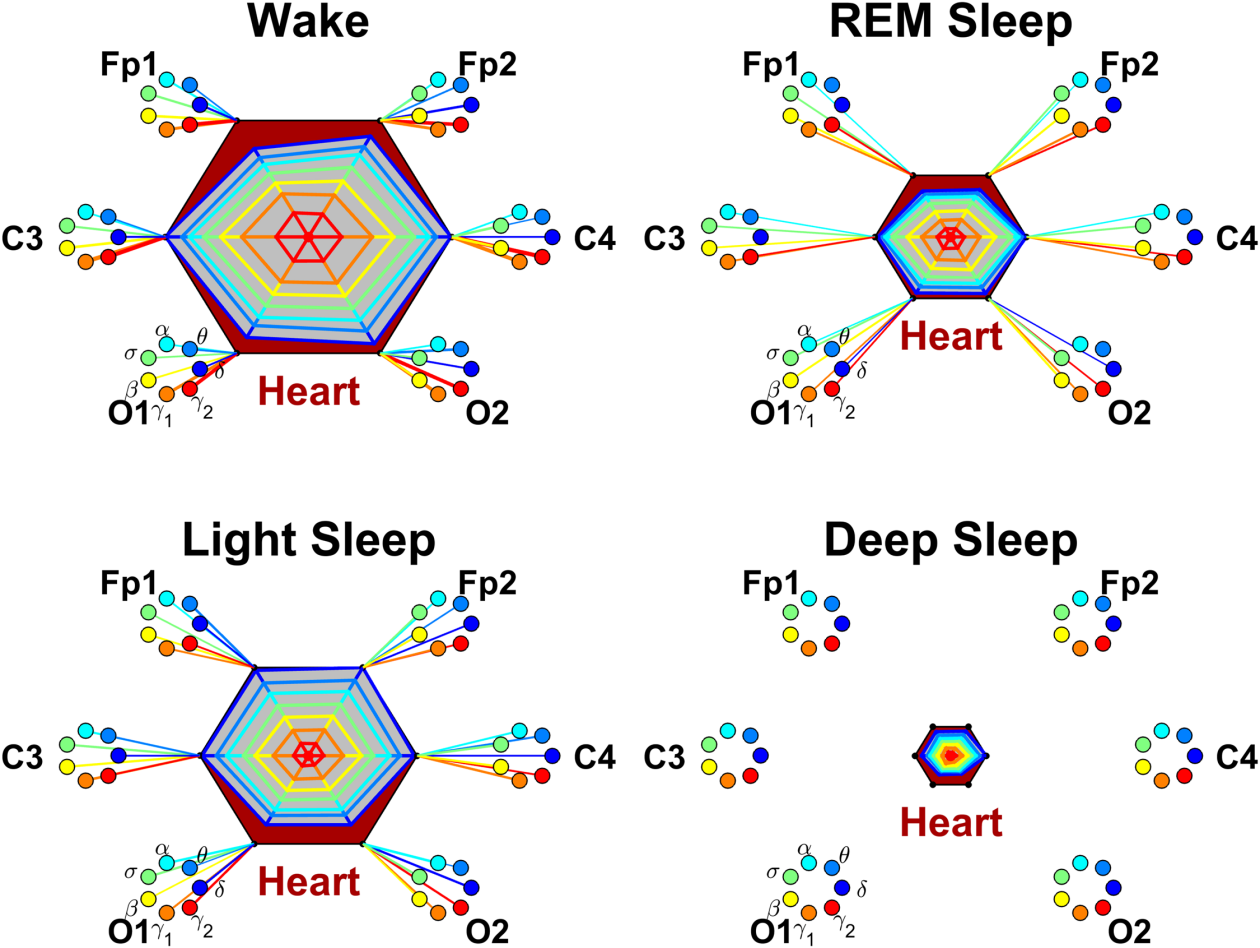
Networks of brain-heart interactions during different physiologic states. Brain areas are represented by Frontal (Fp1 and Fp2), Central (C3 and C4) and Occipital (O1 and O2) EEG channels. Network nodes with different colors represent seven frequency bands (*δ*, *θ*, *α*, *σ*, *β*, *γ*_1_, *γ*_2_) in the spectral power of each EEG channel. Network links between the heart (red hexagon) and EEG frequency nodes at different locations are determined based on the TDS measure (See Section Methods), and links strength is illustrated by the line thickness. Shown are links with strength ≥5%TDS. Radar-charts centered in each hexagon represent the relative contribution of brain control from different brain areas to the strength of network links during different sleep stages. The length of each segment along each radius in the radar-charts represents TDS coupling strength between the heart and each frequency band at each EEG channel location. These segments are shown in the same color as the corresponding EEG frequency nodes. During W and REM, the brain-heart network interactions are mediated mainly through high-frequency *γ*_1_ and *γ*_2_ bands (orange and red links), while during LS and DS, the interactions are mediated uniformly through all frequency bands. In contrast to the brain-eye network (Fig 7), the brain-heart network is characterized by relatively symmetric links strength to all six brain areas, as shown by the symmetric radar-charts in each hexagon. A pronounced stratification pattern is observed for the overall strength of network links—stronger links during W and LS (larger hexagons) and weaker links during REM and DS (smaller hexagons). Notably, compared to the brain-eye (Fig 7), brain-chin (Fig 9) and brain-leg networks (Fig 11), there are no links in the brain-heart network during DS (all links <5%TDS).

**Fig 14:**
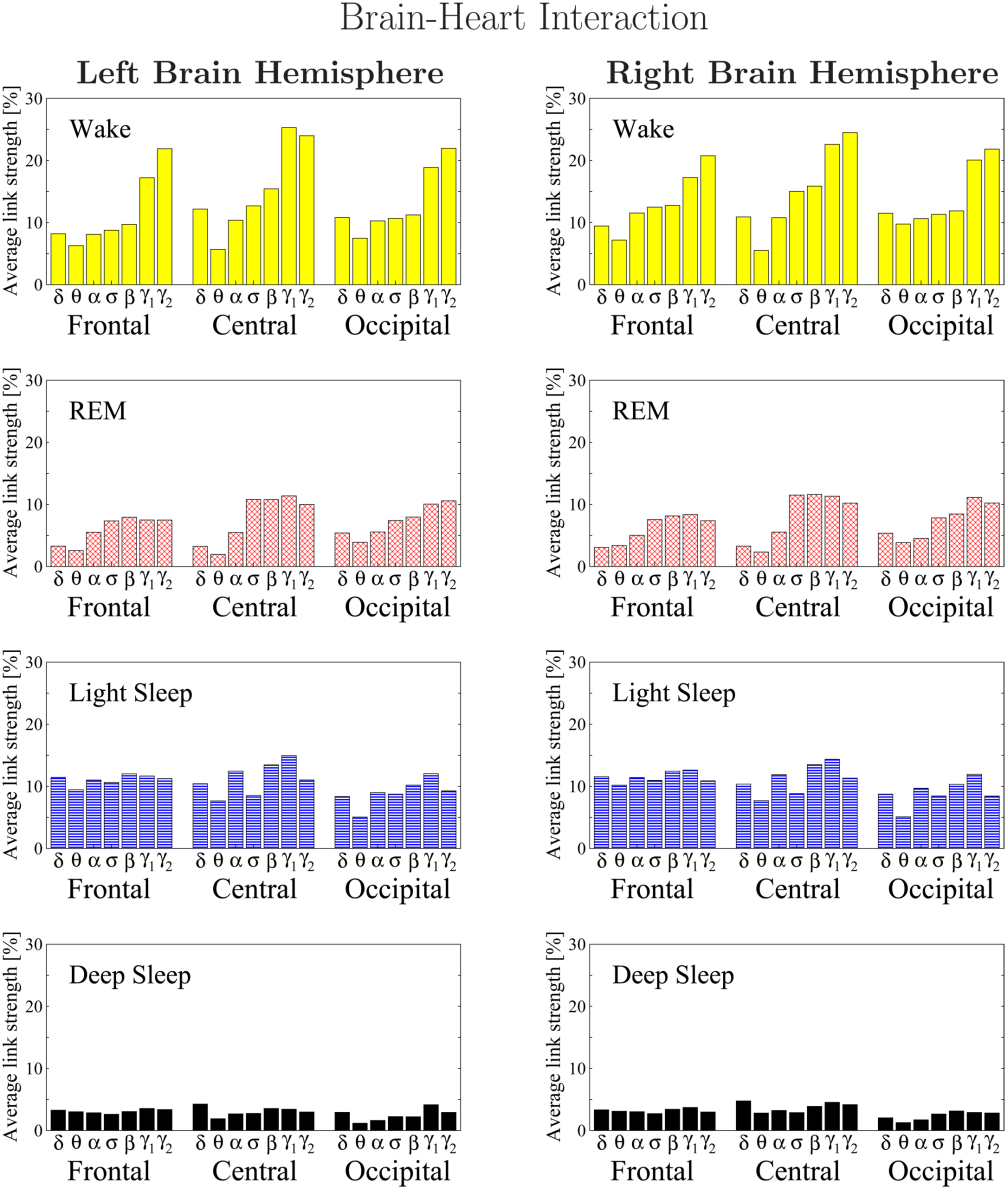
Histograms of links strength in the brain-heart network during different sleep stages. Group averaged links strength are obtained using the TDS measure, where each link represents the interaction of the heart with a given brain area through a specific frequency band. Links are separately grouped by brain areas (Frontal, Central or Occipital), and are arranged in order from low-frequency (*δ* and *θ*) to high-frequency (*γ*_1_ and *γ*_2_) bands. For a given physiologic state, the frequency profile of the TDS links strength of brain-heart interactions remains stable for all brain areas. In contrast to other brain-organ networks where the frequency profile is stable across sleep stages, the frequency profiles of links strength in the brain-heart network change with transitions across different physiologic states—for all brain areas the frequency profiles become flatter as sleep depth increases—indicating that the mechanisms driving brain-heart interactions are markedly different compared to other organs. The sleep-stage stratification observed for the links strength in the brain-heart radar-charts is consistently observed for all frequency bands and brain areas as shown in the histograms—stronger links during W and LS, and weaker links during REM and DS. There is a strong symmetry in the links strength distribution between the left and right hemispheres during all sleep stages.

Our analysis of the network of brain-heart interactions shows a relatively symmetric distribution of the average links strength for different brain areas, with a slight prevalence in strength for the links between the heart and the Central brain areas (C3 and C4), as indicated by the radar chart inside the heart hexagon in Fig 13. We find this spatial symmetry in the average brain-heart link strength to hold for all sleep stages (Fig 14).

Next, we study the frequency profile for the strength of the brain-heart links. We find that for a given physiologic state, the frequency profile of brain-heart links remains stable for all brain areas (Frontal, Central and Occipital) as shown in Fig 14. However, comparing different physiologic states we find markedly different frequency profiles for the strength of brain-heart links. Specifically, during W the frequency profiles for the links to the Frontal, Central and Occipital areas are characterized by strongest links for the highest-frequency *γ*_1_ and *γ*_2_ bands and a gradual decrease in links strength for the lower-frequency bands (*β*, *σ*, *α*, *θ*), followed by a slight kink up in link strength for the lowest-frequency *δ* band. With transition to REM, the frequency profiles for all brain areas are modulated, where the relative difference in links strength between different frequency bands is reduced compared to W, and the shape of the profile changes—stronger links for high-frequency bands (*γ*_1_, *γ*_2_, *β*, *σ*) and much weaker links for low-frequency (*α*, *θ*, *δ*). In contrast, during both LS and DS, we find that for all brain areas (Frontal, Central and Occipital) the frequency profiles of links strength are practically homogeneous with an almost flat distribution across all frequency bands. These frequency profiles for the strength of brain-heart interactions (Fig 14) across different sleep stages are very different from the corresponding frequency profiles of the brain-eye (Fig 8), brain-chin (Fig 10) and brain-leg (Fig 12) networks, indicating that the mechanisms driving brain-heart interactions are markedly different compared to other organs.

Systematically investigating the links strength in the brain-heart network for all 7 frequency bands and different sleep stages, we find that the average link strength for the entire network of brain-heart interactions is highest during W and LS, lower during REM and lowest during DS (Fig 13). Further, this sleep-stage stratification pattern is consistently observed for all three sub-networks representing the Frontal-heart, Central-heart and Occipital-heart links across all frequency bands (Fig 14).

Thus, similar to other organs, our observations show that the strength of all links in the brain-heart network, regardless of brain areas or frequency bands, is modulated in the same way with transitions across sleep stages.

Moreover, we uncover the same symmetry between the left and right brain hemispheres for the strength of all network links across brain areas and frequency bands (Fig 14) as we found for other organ systems.

#### Brain-respiratory interactions

**Fig 15:**
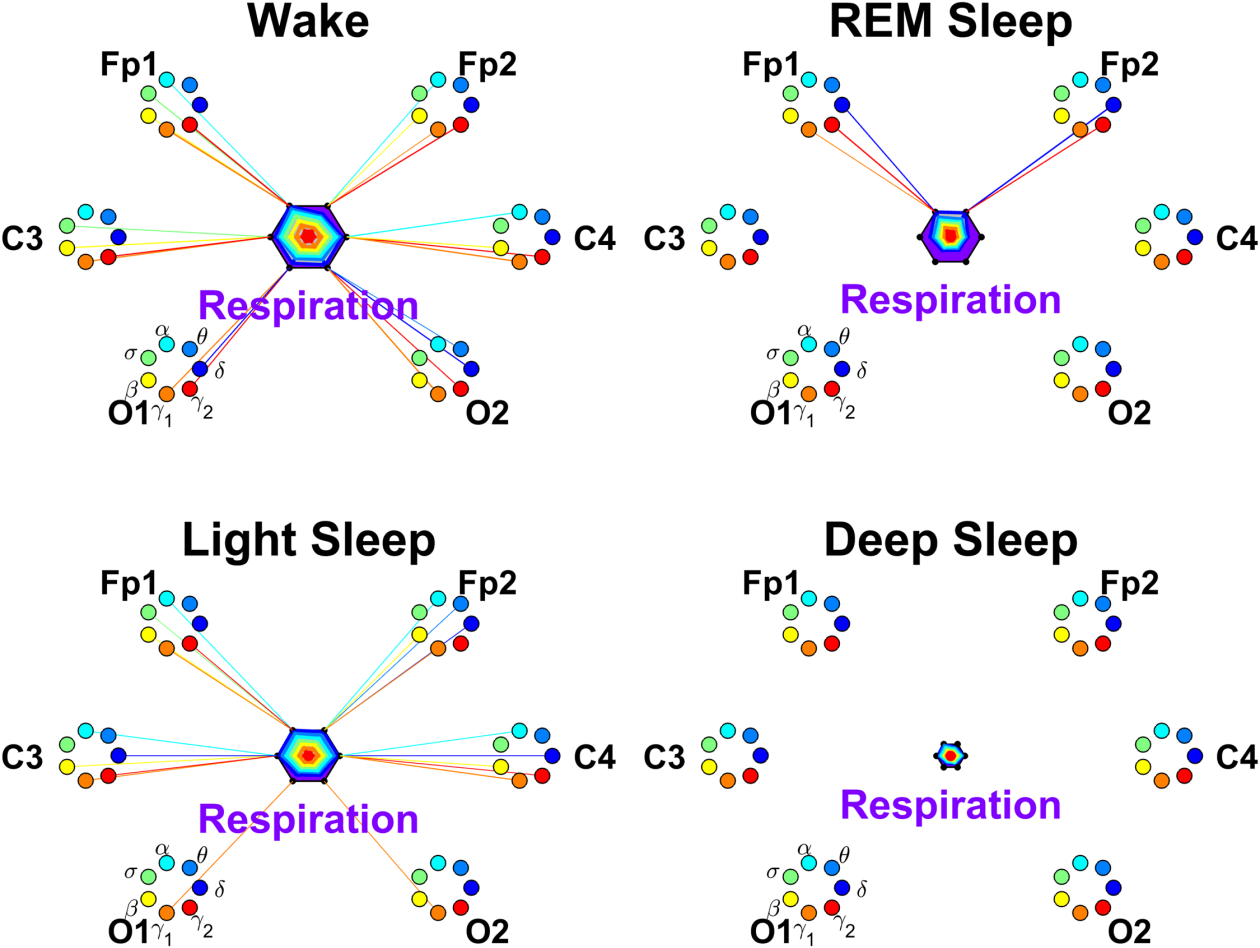
Networks of brain-respiration interactions during different physiologic states. Brain areas are represented by Frontal (Fp1 and Fp2), Central (C3 and C4) and Occipital (O1 and O2) EEG channels. Network nodes with different colors represent seven frequency bands (*δ*, *θ*, *α*, *σ*, *β*, *γ*_1_, *γ*_2_) in the spectral power of each EEG channel. Network links between the respiration (purple hexagon) and EEG frequency nodes at different locations are determined based on the TDS measure (See Section Methods), and links strength is illustrated by the line thickness. Shown are links with strength ≥3%TDS. Radar-charts centered in each hexagon represent the relative contribution of brain control from different brain areas to the strength of network links during different sleep stages. The length of each segment along each radius in the radar-charts represents TDS coupling strength between respiration and each frequency band at each EEG channel location. These segments are shown in the same color as the corresponding EEG frequency nodes. A pronounced stratification pattern is observed for the overall strength of network links—stronger links during W and LS (larger hexagons) and weaker links during REM and DS (smaller hexagons). Note the much weaker links (overall smaller hexagons) for all sleep stages in the brain-respiration network compared to other brain-organ networks, indicating much weaker coupling between the brain and respiration system as measured by %TDS. Similar to the brain-heart network (Fig 13), there are no links in the brain-respiration network during DS (all links <3%TDS).

Finally, we investigate the network of brain-respiratory interactions. Considering the spatial distribution of links strength in the brain-respiratory network, we find a symmetric distribution for links from the Frontal, Central and Occipital brain areas during W, LS and DS. However, during REM, the brain-respiratory interactions are dominant in the Frontal areas, and the links to the Central and Occipital areas are weaker (Fig 15).

**Fig 16:**
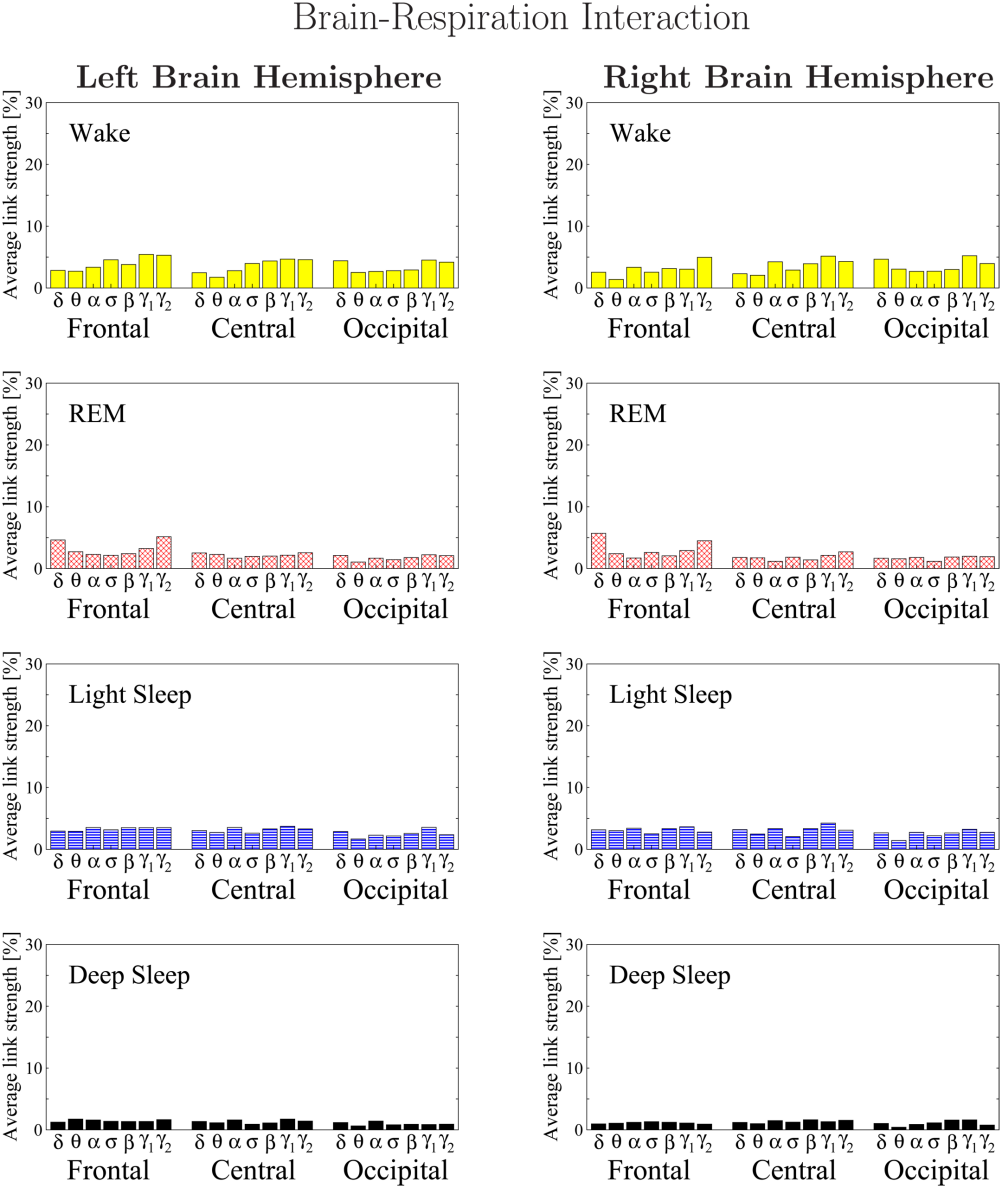
Histograms of links strength in the brain-respiration network during different sleep stages. Group averaged links strength are obtained using the TDS measure, where each link represents the interaction of the respiratory system with a given brain area through a specific frequency band. Links are separately grouped by brain areas (Frontal, Central or Occipital), and are arranged in order from low-frequency (*δ* and *θ*) to high-frequency (*γ*_1_ and *γ*_2_) bands. Brain-respiration networks are characterized by a very homogeneous frequency profile of links strength which is consistently observed across all brain areas—an almost flat distribution across all 7 physiologically relevant frequency bands (*δ*, *θ*, *α*, *σ*, *β*, *γ*_1_, *γ*_2_). The sleep-stage stratification observed for the links strength in the brain-respiration radar-charts (Fig 15) is consistently observed for all frequency bands and brain areas although less pronounce compared to other brain-organ networks. A strong symmetry in the links strength distribution between the left and right hemisphere is present for all sleep stages. Overall brain-respiration links are much weaker than in other brain-organ networks.

Our analysis of the frequency profile for the strength of brain-respiratory links show a very homogeneous pattern with an almost flat distribution across all 7 physiologically relevant frequency bands (*γ*_2_, *γ*_1_, *β*, *σ*, *α*, *θ*, *δ*). This characteristic feature of the frequency profile of the network links strength is present for all brain areas and remarkably remains stable across all sleep stages (Fig 16).

Investigating the links strength in the brain-respiratory network for all frequency bands with focus on difference between sleep stages, we find that the average link strength for the entire network of brain-respiratory interactions is higher during W and LS, lower during REM and lowest during DS (Fig 15).

Further, this sleep-stage stratification pattern is consistently observed for all three sub-networks representing the Frontal-respiratory, Central-respiratory and Occipital-respiratory links across all frequency bands (Fig 16), indicating a modulation with transitions across sleep-stages similar to the one we found for other organ systems. However, we note that for all brain areas and all sleep stages the average strength of the brain-respiratory links is significantly (200–300%) lower compared to the links in the brain-heart and other brain-organ networks.

Comparing the strength of all brain-respiratory links in the left and right brain hemispheres, we find a strong left-right symmetry for all brain areas and frequency bands (Fig 16), a behavior we consistently observed for other organ systems.

#### Integrating all brain-organ interactions

**Fig 17:**
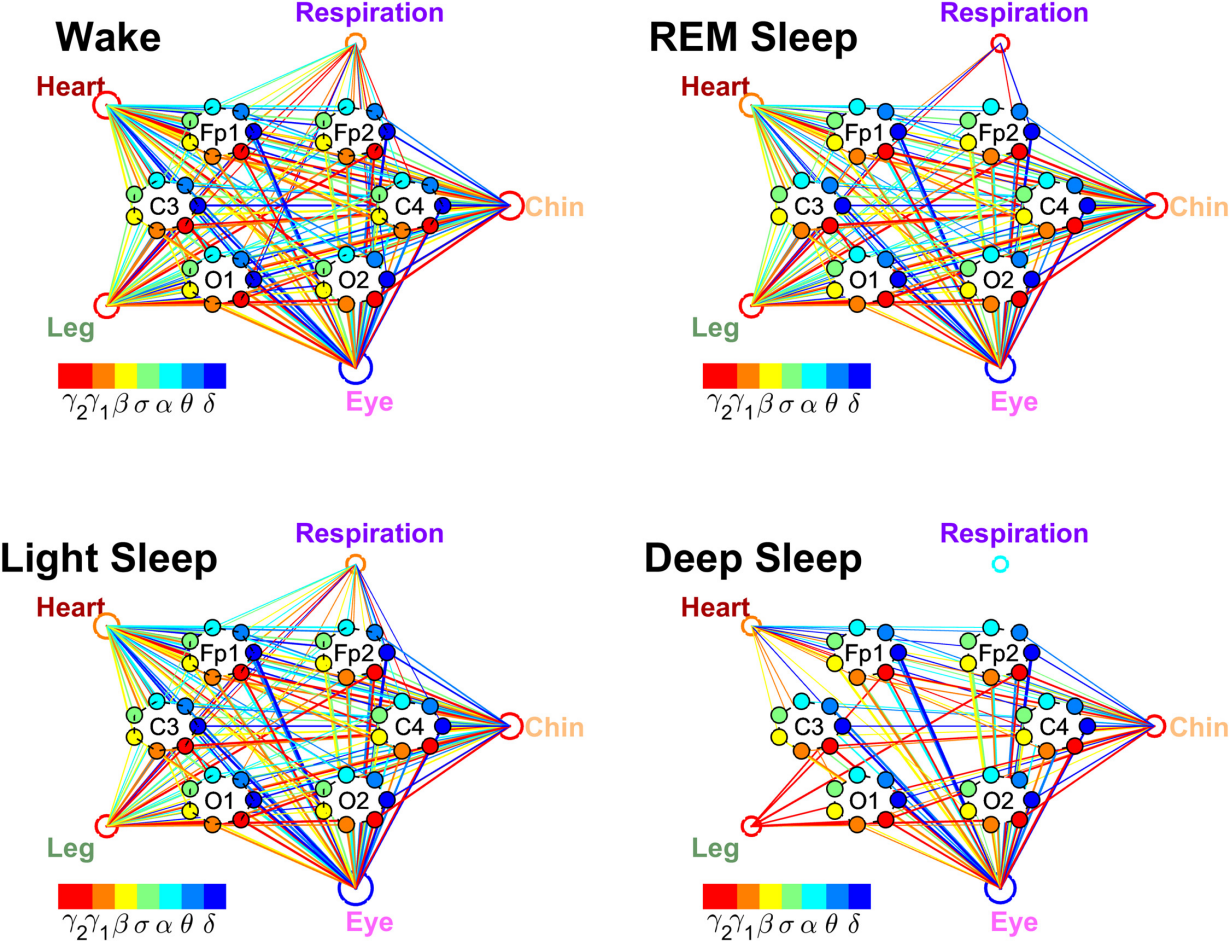
Networks of physiologic interactions between brain areas and key organ systems during different physiologic states. Brain areas are represented by Frontal (Fp1 and Fp2), Central (C3 and C4) and Occipital (O1 and O2) EEG channels. Interactions between brain channels and organ systems are represented by weighted undirected graphs. The size of each organ node in the network is proportional to the strength of the overall brain-organ interaction as measured by the summation of the TDS links strength for all frequency bands and EEG channel locations. The color of each organ node corresponds to the dominant frequency band in the coupling of the organ system with the brain. The width of each link reflects the strength of dynamic coupling as measured by %TDS, and colors of the links correspond to the colors of the nodes representing the different frequency bands (color bars). Plotted are only links with strength ≥3%TDS. Thicker links correspond to stronger coupling and higher time delay stability. The physiological network exhibits transitions across sleep stages—lowest number of links during DS, higher during REM, and highest during LS and W. For different organs, brain-organ interactions are mediated through different dominant frequency bands, e.g., the chin and the leg are predominantly coupled to the brain through the high-frequency *γ*_2_ band during all sleep stages whereas brain-eye network interactions are mediated mainly through low-frequency *δ* band. The complex networks of dynamic interactions between key organ systems and the brain undergoes a hierarchical reorganization across different sleep stages, indicating a previously unknown mechanism of regulation.

After separately investigating the networks of interactions between the brain and different organ systems, we integrate all brain-organ interactions into a single network. This allows us to simultaneously compare several important characteristics of the global network involving brain and multiple organs systems. Specifically, we track the number of links, their strength, the brain areas and frequency bands involved in the interactions between the brain and the group of organ systems and how this global brain-organs network evolves across physiologic states (Fig 17).

This integrative approach makes it possible to compare the predominant frequency band through which the interaction between the brain and different organs are mediated for several organ systems simultaneously during a given physiologic state. We find that the heart, leg and chin always interact with the brain mainly through the high-frequency *γ*_1_ and *γ*_2_ bands (red colored links in Fig 17), whereas the brain-eye interactions are mediated through lowest-frequency *δ* band (blue colored links in Fig 17). We note that there is no single dominating frequency for the brain-respiration interaction, and that the interaction between brain and the respiratory system is always weaker than other brain-organ interactions, indicating a relatively weak physiologic coupling between brain and respiration compared to other organs at the time scales (>2.5 min) over which our TDS analysis is performed.

Further, with transitions across sleep stages we observe a complex hierarchical reorganization in both the number and the strength of links in the integrated brain-organs network—lowest number of links during DS (sparse network), higher during REM, and highest number of links involving most of the frequency bands during LS and W. Remarkably, this structural reorganization of the integrated brain-organs network is consistent with the sleep-stage stratification patterns observed for each individual organ system, indicating a previously unknown general rule of neural regulation of organ systems.

### Organ-Organ Networks

**Fig 18:**
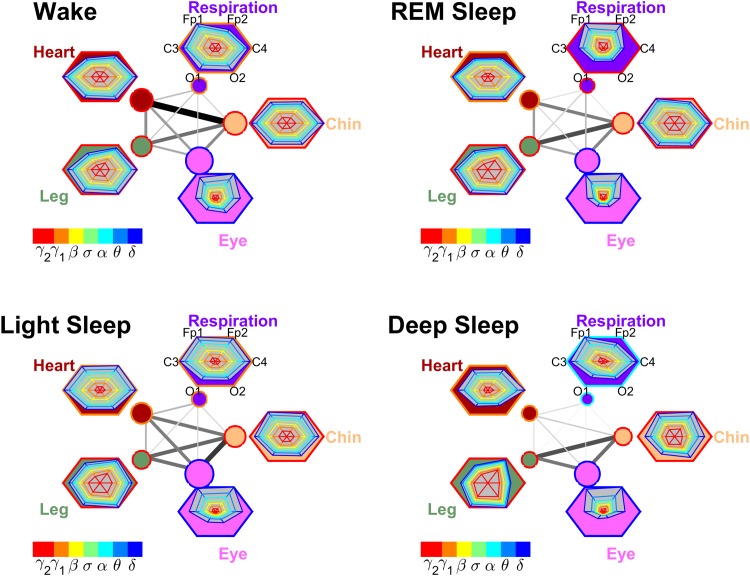
Networks of physiologic interactions among key organ systems during different physiologic states. Interactions among organ systems are represented by weighted undirected graphs, where links reflect the strength of dynamic coupling as measured by % TDS (Section Methods). Darker and thicker links between organ systems correspond to stronger interaction with higher %TDS. The size of each organ node in the network is proportional to the strength of the overall brain-organ interaction as measured by the summation of the TDS links strength for all frequency bands and EEG channel locations. Hexagons representing individual organs in the networks are obtained in the same way as in Figs 7, 9, 11, 13, and 15; and are normalized to the same size. Color bar represents different physiologically relevant frequency bands in the EEG spectral power and is used in the radar-charts for the brain-organ interactions shown in each hexagon. The color of each organ node as well as the edge color of the organ hexagon corresponds to the dominant frequency band in the coupling of the organ system with the brain. Notably, larger organ nodes representing stronger brain-organ interactions are consistently connected by stronger organ-organ links (thicker and darker lines). A pronounced re-organization in the configuration of network links strength is observed with transitions from one sleep stage to another, demonstrating a clear association between network structure and physiologic function.

We develop a novel approach to analyze and graphically present the complex behavior of organ-organ interactions. Integrating information obtained from our investigation of brain-organ interactions, we focus on how organ-to-organ interactions are influenced by neural regulation through different brain areas. We combine radar-charts representing the characteristics of brain-organ interactions with the network of links between all organ systems obtained thought TDS analysis of the output signals for each pair of organ systems (Fig 18).

We observe that, with transitions from one sleep stage to another there is a pronounced structural re-organization in the topology and links strength of the organ-organ network. This demonstrates a clear association between organ-to-organ network structure and physiologic function of the entire organism. The result in Fig 18 shows how physiologic states influence the dynamics of horizontal integration among organ systems through changing the configuration of links strength in the organ-to-organ network.

Specifically, we find that eye, chin and leg are always strongly connected despite the very different characteristics in their interactions with the brain—i.e., different dominating frequency bands (different color rim of the organ hexagons) and different involvement of the brain areas (different shape of radar-charts in each hexagon) and different overall strength of their network interaction with the brain (different size of the nodes representing each organ), as shown in Fig 18. In contrast, the heart and respiratory system significant vary their degree of coupling with the rest of network across physiologic states (Fig 18). Further, we note that even when two organ systems predominately interact with the same brain areas, their coupling strength in the organ-to-organ network can still exhibit a complex transition across different physiologic state—for example, both heart and chin which predominately interact with Central brain areas, however, the strength of the heart-chin link in the organ network dramatically change across different sleep stages.

Interestingly, we discover that strong organ-to-organ links often occur between large nodes in the network that represent strong brain-organ interactions, suggesting our TDS network approach captures significant cerebral component in organ-organ interactions. Notably, the reduced link strength of the heart and respiratory system in the organ-to-organ network during LS and DS compared to REM and W is consistent with earlier findings of reduced sympathetic input and corresponding loss of long-range auto-correlation in cardiac and respiratory dynamics during LS and DS [22–25].

## Discussion

In summary, we develop a novel analytical method based on the concept of Time Delay Stability, which allows us to identify and quantify network interactions between diverse physiologic systems with very different types of dynamics over a broad range of time scales and where their complex output signals continuously change in response to transitions across physiologic states. By investigating the dynamics of synchronous bursts of activations in neurophysiologic output signals from diverse organ systems we quantify their coupling, and we study dynamical links among systems under different physiologic states. Integrating organ-to-organ interactions into a physiologic network, we are able to probe for the first time how organ systems coordinate and optimize their function to produce distinct physiologic states.

We further develop a novel visualization approach to transform dynamical interactions among organs into network graphs that simultaneously capture several fundamental aspects of the complexity and nature of physiologic coupling. Combining the TDS method and the visualization approach, we obtain first dynamic maps of organ network interactions.

We find that during different physiologic states, the network of organ-to-organ interactions is characterized by different configurations of links and links strength. In addition, we observe that with transition from one physiologic state to another the network of interactions among organ systems undergoes a fast hierarchical reorganization that occurs on time scale from seconds to minutes, indicating a rapid dynamical response to physiologic changes. Our results are first demonstration of direct association between physiologic network topology and physiologic function.

Our investigations led to the discovery of several basic rules of regulation that underlie network dynamics of organ interactions:

1. The brain-brain network is a complex two-layered dynamical network that consists of: (i) an intra-channel sub-network of local (within an EEG channel) communications between different physiologically relevant brain-wave frequency bands, and (ii) an inter-channel sub-network of interactions across different brain areas (Frontal, Central and Occipital) mediated through various frequency bands.
  With transitions across physiologic states such as different sleep stages, the brain-brain network undergoes a hierarchical reorganization through a well-structured process involving several building blocks of network configurations. We find that each of these building blocks involves specific types of links, e.g. links across different brain areas mediated by the same frequency band or links between different frequency bands.
  Further, we find that the strength of brain-brain network links follows a robust rank order: (a) within a given brain hemisphere, Frontal-Central links are stronger than Central-Occipital, which are stronger than Frontal-Occipital links; (b) across the left and right brain hemispheres, Frontal-Frontal links are stronger than Central-Central, which are stronger than Occipital-Occipital. These rank order is preserved for each sleep stage.
  Our statistical analysis of the building blocks in the network of brain-brain interactions reveals a pronounced stratification pattern across sleep stages, i.e. higher network connectivity and average links strength during W and LS, and much lower connectivity and links strength during REM and DS.
2. Networks of brain-organ interactions reveal different involvement of Frontal, Central and Occipital brain areas the regulation of organ systems, and that these network interactions are predominantly mediated through specific frequency bands.
  While the brain-eye networks exhibit strongest links to the Frontal areas, other brain-organ networks are characterized by a spatially symmetric distribution in the coupling strength with different brain areas. Notably, this spatial distribution of coupling strength to different brain areas changes significantly for certain organ systems with transitions across different sleep stages, e.g. brain-respiration network, whereas other brain-organ networks exhibit stable spatial distribution of network links strength to different brain areas.
  We discover that in its communication with the brain each organ has its own frequency profile, representing the relative strength of brain-organ links mediated through the different frequency bands. For some organs, these frequency profiles are characterized by the presence of a dominant frequency—e.g., strongest links in the brain-leg network are mediated through the highest-frequency *γ* band, while the strongest links in the brain-eye network are mediated through the lowest-frequency *δ* band. In contrast, other organ systems have relatively uniform (flat) frequency profiles such as the brain-respiratory network.
  For all brain-organ networks the organ-specific frequency profiles are consistently observed for each subgroup of network links to the Frontal, Central and Occipital brain areas. Further, we find that these frequency profiles remain stable with transitions across sleep stages (with the exception of brain-heart network).
3. For both brain-brain and brain-organ networks, we find a remarkable symmetry between the left and right brain hemispheres in both network topology and the configuration of links strength.
4. We find a very robust sleep-stage stratification pattern for all brain-brain, brain-organ and organ-organ networks—more and stronger links during W and LS, and less and weaker links during REM and DS. Further, in all these networks, the sleep-stage stratification is consistently observed for all subgroups of network links related to the Frontal, Central and Occipital areas. Moreover, this sleep-stage stratification pattern is stable even when we consider only links that are medicated through a single frequency band.
5. Our results demonstrate a direct association between network topology and physiologic function. We uncover new basic principles of how physiologic networks reorganize in response to well defined physiologic states. The network behavior we find is universal, since it is observed for every healthy subject in the database we analyzed, and thus points to a new previously unknown regulatory mechanism that underlies the dynamics of organ interactions.

These findings are first steps in understanding how organ systems synchronize and coordinate their output dynamics as a network to produce distinct physiologic functions. Our investigations reveal basic rules that underlie (i) the dynamics of network interactions among organ systems, and (ii) the hierarchical reorganization of organ network interactions in response to changes in physiologic state. To our knowledge, this is the first report on how an entire network of diverse dynamical systems hierarchically reorganizes its structure in real time to facilitate distinct functions. The presented here findings and visualization maps are initial steps in building the first atlas of dynamic interactions among organ systems.
